# Immune checkpoint inhibitors related respiratory disorders in patients with lung cancer: A meta-analysis of randomized controlled trials

**DOI:** 10.3389/fimmu.2023.1115305

**Published:** 2023-02-28

**Authors:** Han Liu, Sean X. Luo, Jing Jie, Liping Peng, Shuai Wang, Lei Song

**Affiliations:** ^1^Department of Respiratory Medicine, The First Hospital of Jilin University, Changchun, Jilin, China; ^2^Department of Vascular Surgery, General Surgery Center, The First Hospital of Jilin University, Changchun, Jilin, China

**Keywords:** immune checkpoint inhibitors (ICI), respiratory disorders, lung cancer, meta-analysis, randomized controlled trials (RCT)

## Abstract

**Background:**

In recent years, immune checkpoint inhibitors (ICIs) had extremely rapid growth in anti-cancer and improved outcomes of many malignancies, specifically lung cancer. However, the incidence of ICIs-related adverse events also raised. Using this meta-analysis, ICIs-related respiratory disorders were investigated in lung cancer patients.

**Methods:**

Using Cochrane Library, Embase, and PubMed databases, we performed an integrated search for randomized controlled trials (RCTs) to compare respiratory disorders among different regimens. The data was prepared with the Preferred Reporting Items for Systematic Reviews and Meta-Analyses (PRISMA) reporting guideline, and the quality of included studies was evaluated based on the Cochrane manual.

**Results:**

In total, 22 RCTs were involved in this meta-analysis. Compared with ICIs, chemotherapy reduced the risk of interstitial lung disease (p = 0.03; SMD: 2.81; 95% CI: 1.08, 7.27), pleural effusion (p = 0.002; SMD: 2.12; 95% CI: 1.32, 3.42), and pneumonitis (p < 0.00001; SMD: 9.23; 95% CI: 4.57, 18.64). ICIs plus chemotherapy could provide a higher probability for patients to suffer pneumonitis than chemotherapy (p = 0.01; SMD: 1.96; 95% CI: 1.17, 3.28). In addition, single ICI brought a lower likelihood for patients suffering pneumonitis than double ICIs (p = 0.004; SMD: 2.17; 95% CI: 1.27, 3.69).

**Conclusion:**

ICIs-based treatment, such as ICIs alone, ICIs plus chemotherapy and double ICIs, can raise the incidences of some respiratory disorders in patients with lung cancer. It suggests that ICIs should be conducted based on a comprehensive consideration to prevent ICIs-related respiratory disorders. To a certain degree, this study might be provided to the clinician as a reference for ICIs practice.

**Systematic review registration:**

https://www.crd.york.ac.uk/prospero/display_record.php?ID=CRD42022378901, identifier (CRD42022378901).

## Introduction

In most countries, cancer is currently the first or second most frequent cause of premature death. In 2022, the USA has experienced more than 1,900,000 new cancer cases and 600,000 cancer deaths, with lung cancer being the leading cause of these deaths (1). Fortunately, the survival rate of patients with lung cancer has improved, which may be related to the early screening of lung cancer. Furthermore, a significant progress in non-small cell lung cancer (NSCLC) treatment with the advent of targeted drugs, coupled with the approval of immunotherapy by the Food and Drug Administration (FDA) in 2015, has also contributed to the population-level improvement in lung cancer-specific survival (2).

Many treatments to control malignancies by mobilizing the immune system are under investigation, including cytokines, T cells (checkpoint inhibitors, co-stimulatory receptor agonists), T cell engineering, oncolytic viruses, and vaccines. Immune checkpoint inhibitor (ICI) therapy includes programmed death-1 (PD-1) and programmed cell death 1 ligand 1/2 (PD-L1/2), cytotoxic T lymphocyte-associated antigen-4 (CTLA-4), lymphocyte-activation gene 3 (LAG3), and other potential targets. PD-1 is a transmembrane protein expressed in T, B, and NK cells and an inhibitory molecule that binds to PD-L1 and PD-L2. PD-L1 is represented on the cell surface of various tissue types, including many tumor and hematopoietic cells. Contrarily, PD-L2 is more restricted to hematopoietic cells. The combination of PD-1 and PD-L1/2 can directly inhibit tumor cell apoptosis and promote peripheral effector T cell depletion and conversion of effector T cells into Treg cells (3, 4). To date, the results of many large-scale randomized controlled trials (RCTs) of PD-1 inhibitors against lung cancer have confirmed the concept of durable antitumor responses and improved progression-free survival and overall survival (OS) (5). CTLA-4 was recognized as a negative regulator of T cell activation in the mid-1990s (6–8). CTLA-4 on the surface of CD4+ and CD8+ T cells can play a role by binding to the co-stimulatory receptors CD80 and CD86 on the surface of APCs with a higher affinity than the co-stimulatory receptor CD28 on the surface of T cells (9). Scientists believe CTLA-4 to be APC-triggered, acting as a brake on CD4+ and CD8+ T cell activation. LAG3 is expressed on B cells, specific T cells, NK cells, and tumor-infiltrating lymphocytes, where it regulates immune checkpoint pathways (10). With the deepening of the understanding of the immune mechanism, several other potential targets of immune checkpoint inhibition have been discovered, one after another, such as B and T lymphocyte attenuator, V-domain Ig suppressor of T cell activation, T cell immunoglobulin, and mucin domain-3. ICIs have become first-line treatments for various malignancies, with the addition of immunotherapy to surgery, radiotherapy, chemotherapy, and targeted therapy (11, 12).

Despite the favorable clinical benefits of checkpoint inhibition, it has side effects known as immune-related adverse events (irAEs) (13, 14). IrAEs include skin diseases, diarrhea, hepatotoxicity, and cardiotoxicity (15–19). Checkpoint inhibition may also cause fulminant or fatal toxic reactions (20). However, there is no comparative research to comprehensively discuss respiratory disorders caused by ICIs in lung cancer. Thus, we conducted this meta-analysis to identify potential respiratory diseases during ICI therapy for lung cancer to guide the selection of patients who should benefit from ICIs.

## Materials and methods

### Reporting standards

The meta-analysis for ICIs-related respiratory disorders in patients with lung cancer was prepared in accordance with the Preferred Reporting Items for Systematic Reviews and Meta-analyses reporting guideline (21).

### Search strategy

A competent information specialist (HL) conducted an integrated search for RCTs between January 2000 and October 2022 using the Cochrane Library, Embase, and PubMed databases. According to the PICOS (participants, interventions, comparisons, outcomes, and study design) guidelines (22), “ICIs,” “PD-1,” “PD-1 inhibitors,” “PD-L1,” “PD-L1 inhibitors,” “CTLA-4,” “CTLA-4 inhibitor,” “atezolizumab,” “avelumab,” “camrelizumab,” “cemiplimab,” “durvalumab,” “ipilimumab,” “nivolumab,” “pembrolizumab,” “sintilimab,” “tislelizumab,” “toripalimab,” “tremelimumab,” “lung cancer,” “lung carcinoma,” “neoplasms,” “adverse reactions,” “adverse events,” and “randomized controlled trial” were entered as the Medical Subject Heading terms.

### Inclusion and exclusion criteria

The inclusion criteria were as follows: (1) RCT on lung cancer (phase II or III clinical trials); (2) ICI intervention, including PD-1/PD-L1 or CTLA-4 inhibitors; and (3) comparison between single-agent ICI plus chemotherapy and chemotherapy, single-agent ICI and chemotherapy, as well as single-agent and double-agent ICIs. The exclusion criteria were as follows: (1) no report of ICIs-related respiratory disorders; (2) publications not written in English; (3) abstracts, case reports, comments, editorials, letters, and reviews; and (4) duplicate, missing, and overlapping datasets.

### Study selection

Two investigators (SL and JJ) independently reviewed the titles and abstracts of the articles to obtain the qualified studies. Furthermore, two investigators (SW and LS) identified the potentially relevant studies to determine if they were eligible based on the inclusion and exclusion criteria. Disagreements as regards the study’s selection were resolved through discussion and compromise.

### Data extraction

**Figure 1:**
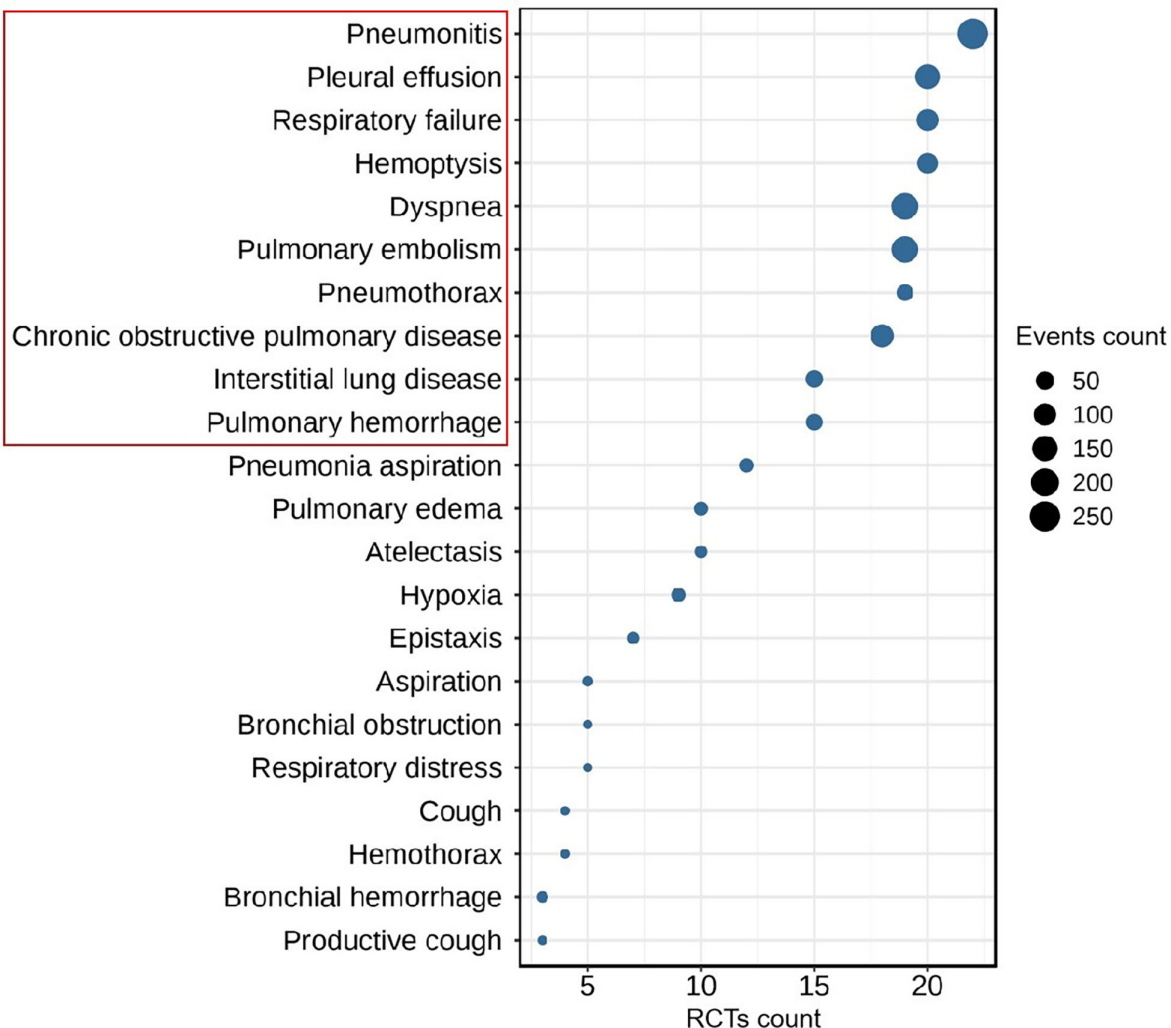
The Top-10 most frequent ICIs-related respiratory disorders in patients with lung cancer (RCTs count ≥ 3, event count ≥ 5).

Two investigators (HL and JJ) independently extracted the characteristic data, including publication year, first author name, number of clinical trial, drug name, clinical trial phase, lung cancer type, regiment of intervention, enrollment, and serious adverse events (SAEs), from the eligible studies. According to the analysis of SAEs, the top 10 most frequent ICIs-related respiratory disorders, including pneumonitis, dyspnea, pulmonary embolism, pleural effusion, chronic obstructive pulmonary disease, respiratory failure, hemoptysis, interstitial lung disease, pulmonary hemorrhage, and pneumothorax, were conducted as the main outcomes (**Figure 1**). Disagreements as regards data extraction were resolved through discussion and compromise.

### Quality assessment

Based on the Cochrane manual, the bias risk of eligible studies, including allocation concealment, blinding of participants and personnel, blindness to outcome assessment, incomplete outcome data, random sequence generation, selective outcome reporting, and other bias, was independently evaluated by two investigators (SW and SL) (23). Funnel plot were performed to assess publication bias (24). Disagreements regarding quality assessment were resolved through discussion and compromise.

### Data synthesis and statistical analysis

All statistical analyses were calculated using Review Manager (RevMan v5.3). ICIs-related respiratory disorders, including pneumonitis, dyspnea, pulmonary embolism, pleural effusion, chronic obstructive pulmonary disease, respiratory failure, hemoptysis, interstitial lung disease, pulmonary hemorrhage, and pneumothorax, were evaluated using the mean differences with 95% confidence intervals (CIs). Based on the Cochrane collaboration network, a fixed-effects model was used to pool studies, and the inconsistency index was used to access heterogeneity as low (*I^2^* < 30%), moderate (30% ≤ *I^2^* < 50%), or high (*I^2^* ≥ 50%) (25, 26). Subanalyses of the effects of the different intervention on ICIs-related respiratory disorders were conducted. A two-tailed *P*-value of <0.05 was considered statistically significant.

## Results

### Study selection

**Figure 2:**
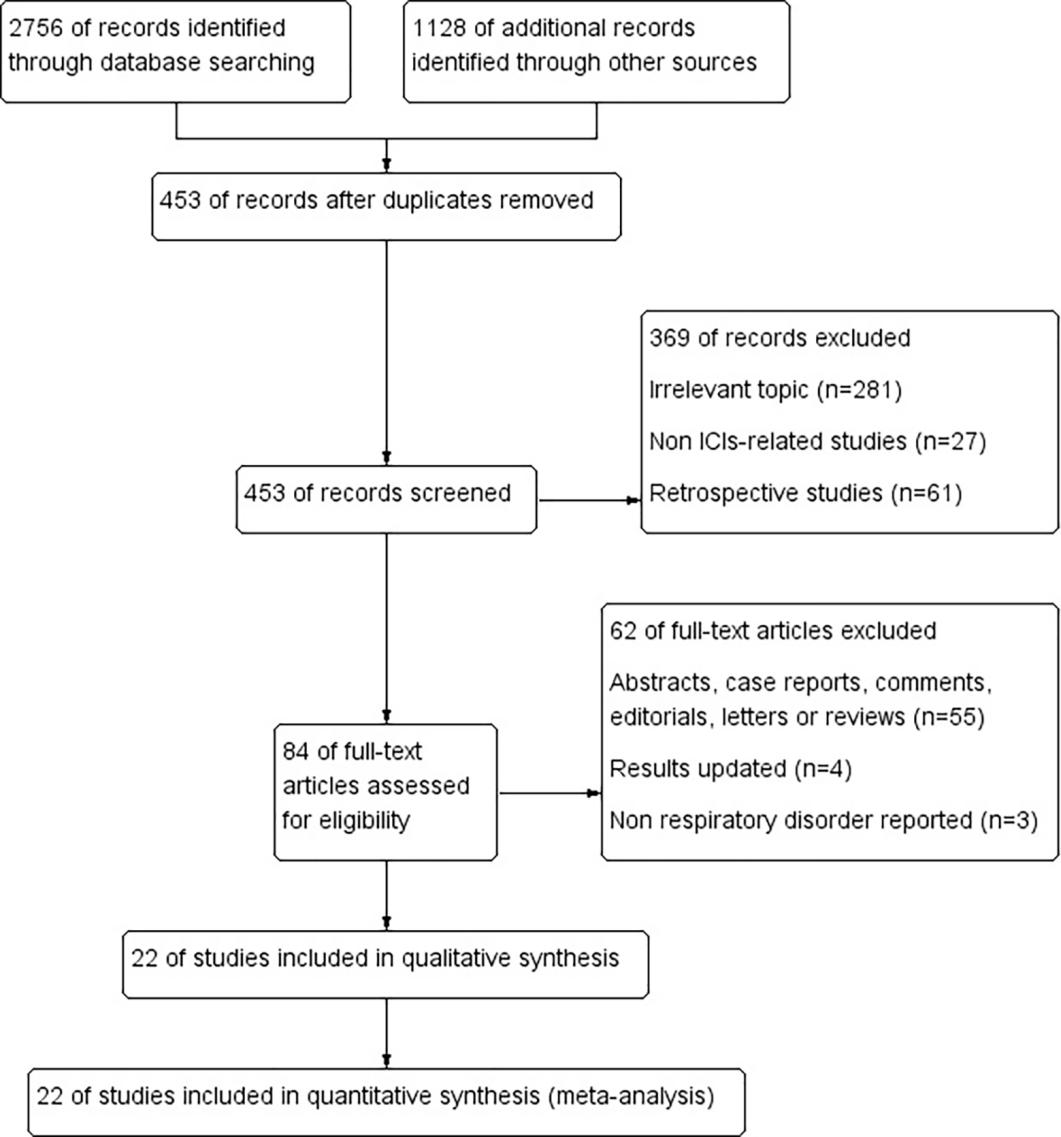
The flow diagram of the study identification and selection process.

In total, 3884 potentially relevant studies were retrieved from databases as a result of the strategy performed in searching. 453 studies were recorded after duplicates removed. Then, 369 studies were excluded due to irrelevant topic, non ICIs-related or retrospective studies. The investigators removed 62 studies after screening the full texts. Four released updated results (27–30), and three did not report respiratory disorders (31–33). Finally, 22 studies were included as the flow diagram described in **Figure 2** (34–55).

### Study characteristics

The characteristics of 22 RCTs from the 22 included studies are presented in **Table 1**. The studies ranged in year of publication from 2015 to 2021. Enrollment of 22 RCTs was 11460. Eighteen RCTs included NSCLC and four included small cell lung cancer (SCLC). PD-1 inhibitors were administered in 16 RCTs, PD-L1 inhibitors in 6, and CTLA-4 inhibitors in 4. In the treatment regimens, 9 RCTs were conducted to compare ICIs plus chemotherapy vs. chemotherapy, 10 RCTs about ICIs vs. chemotherapy, and 3 RCTs about double ICIs vs. single ICI.

**Table 1:** Characteristics of 22 studies included in analysis of ICIs-related respiratory disorders.

NO.	First Author Publication Year	Clinical Trial	Trial Phase	Cancer Type	Drug	Treatment Regimens	Enrollment
1	Arrieta, 2020 (34)	PROLUNG (NCT02574598)	II	NSCLC	Pembrolizumab (PD-1)	ICIs + Chemotherapy VS. Chemotherapy	78
2	Awad, 2021 (35)	KEYNOTE-021 (NCT02039674)	II	NSCLC	Pembrolizumab (PD-1)	ICIs + Chemotherapy VS. Chemotherapy	121
3	Barlesi, 2018 (36)	JAVELIN Lung 200 (NCT02395172)	III	NSCLC	Avelumab (PD-L1)	ICIs VS. Chemotherapy	758
4	Borghaei, 2015 (37)	CheckMate057 (NCT01673867)	III	NSCLC	Nivolumab (PD-1)	ICIs VS. Chemotherapy	555
5	Boyer, 2021 (38)	KEYNOTE-598 (NCT03302234)	III	NSCLC	Pembrolizumab (PD-1) Ipilimumab (CTLA-4)	Double ICIs VS. single ICI	563
6	Brahmer, 2015 (39)	CheckMate 017 (NCT01642004)	III	NSCLC	Nivolumab (PD-1)	ICIs VS. Chemotherapy	260
7	Carbone, 2017 (40)	CheckMate 026 (NCT02041533)	III	NSCLC	Nivolumab (PD-1)	ICIs VS. Chemotherapy	530
8	Gadgeel, 2020 (41)	KEYNOTE-189 (NCT02578680)	III	NSCLC	Pembrolizumab (PD-1)	ICIs + Chemotherapy VS. Chemotherapy	607
9	Gettinger, 2021 (42)	Lung-MAP 1400l (NCT02785952)	III	NSCLC	Nivolumab (PD-1) Ipilimumab (CTLA-4)	Double ICIs VS. single ICI	247
10	Goldman, 2021 (43)	CASPIAN (NCT03043872)	III	SCLC	Durvalumab (PD-L1) Tremelimumab (CTLA-4)	ICIs + Chemotherapy VS. Chemotherapy	531
11	Herbst, 2020a (44)	IMpower110 (NCT02409342)	III	NSCLC	Atezolizumab (PD-L1)	ICIs VS. Chemotherapy	549
12	Herbst, 2020b (45)	KEYNOTE-010 (NCT01905657)	III	NSCLC	Pembrolizumab (PD-1)	ICIs VS. Chemotherapy	991
13	Horn, 2018 (46)	IMpower133 (NCT02763579)	III	SCLC	Atezolizumab (PD-L1)	ICIs + Chemotherapy VS. Chemotherapy	394
14	Mok, 2019 (47)	KEYNOTE-042 (NCT02220894)	III	NSCLC	Pembrolizumab (PD-1)	ICIs VS. Chemotherapy	1251
15	Nishio, 2021 (48)	IMpower132 (NCT02657434)	III	NSCLC	Atezolizumab (PD-L1)	ICIs + Chemotherapy VS. Chemotherapy	565
16	Owonikoko, 2021 (49)	CheckMate 451 (NCT02538666)	III	SCLC	Nivolumab (PD-1) Ipilimumab (CTLA-4)	Double ICIs VS. single ICI	557
17	Paz-Ares,2020 (50)	KEYNOTE-407 (NCT02775435)	III	NSCLC	Pembrolizumab (PD-1)	ICIs + Chemotherapy VS. Chemotherapy	558
18	Reck, 2019 (51)	KEYNOTE-024 (NCT02142738)	III	NSCLC	Pembrolizumab (PD-1)	ICIs VS. Chemotherapy	304
19	Spigel, 2021 (52)	CheckMate 331 (NCT02481830)	III	SCLC	Nivolumab (PD-1)	ICIs VS. Chemotherapy	547
20	West, 2019 (53)	IMpower130 (NCT02367781)	III	NSCLC	Atezolizumab (PD-L1)	ICIs + Chemotherapy VS. Chemotherapy	604
21	Wu, 2019 (54)	CheckMate 078 (NCT02613507)	III	NSCLC	Nivolumab (PD-1)	ICIs VS. Chemotherapy	493
22	Yang, 2020 (55)	Orient-11 (NCT03607539)	III	NSCLC	Sintilimab (PD-1)	ICIs + Chemotherapy VS. Chemotherapy	397

### Quality assessment

**Figure 3:**
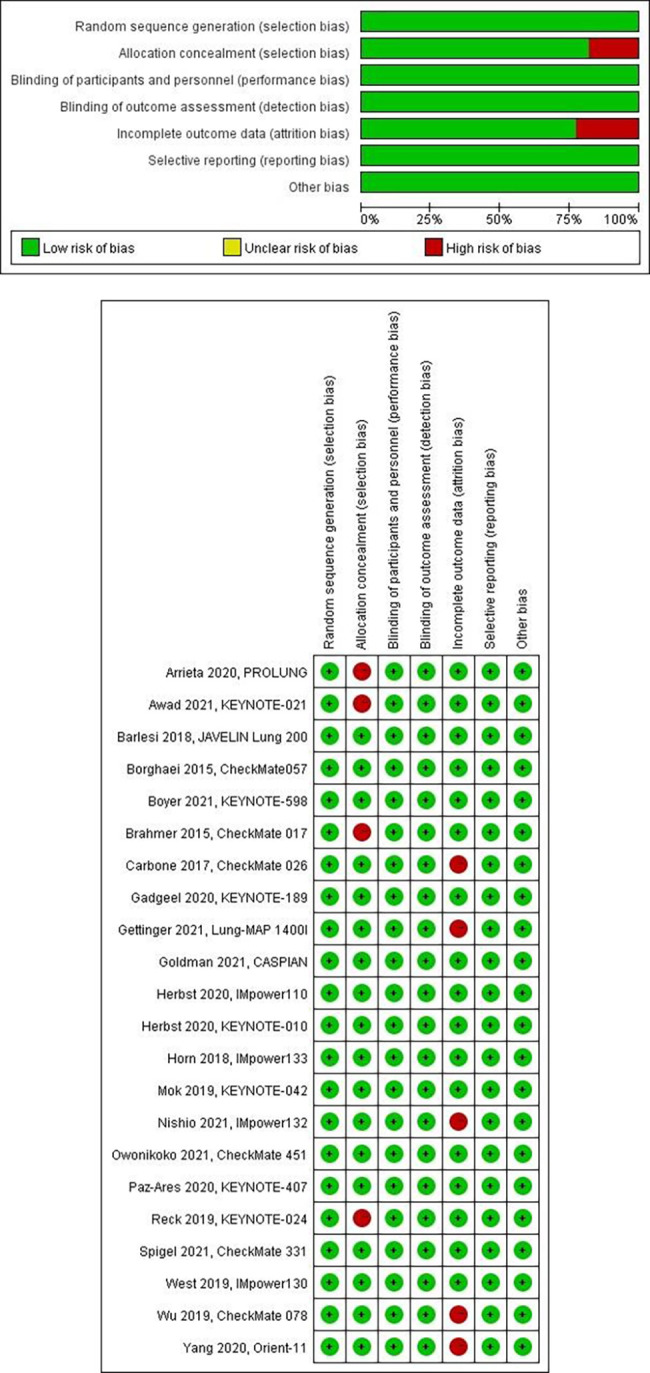
Assessment of bias risk, **(A)** risk of bias graph, **(B)** risk of bias summary.

The quality assessment of the included studies is presented in **Figure 3**. In sum, all studies were randomized, with five presenting a high risk of bias in allocation concealment (selection bias). All studies showed low risks of blinding of participants and personnel (performance bias), blinding of outcome assessment (detection bias), and selective reporting (reporting bias), with five studies demonstrating high risks of incomplete outcome data (attrition bias). Overall, the risk of other bias was low. The potential publication bias was evaluated through visual inspection of a funnel plot (**Supplementary material**).

### Risk of ICIs-related respiratory disorders

The incidence of ICIs-related respiratory disorders in different treatment regimens is presented in **Table 2**. In total, the incidence rates of pneumonitis (2.14%), dyspnea (1.62%), and pulmonary embolism (1.51%) were the three highest than other respiratory disorders. Especially in double ICI treatment regimens, the incidence rates of pneumonitis and dyspnea were 6.43% and 3.65%, respectively. Compared with chemotherapy treatment regimens, the incidence rates of pneumonitis, interstitial lung disease, and pleural effusion increased by more than two-fold in ICI treatment regimens (2.84% vs. 0.22%, 0.69% vs. 0.15%, 1.68% vs. 0.83%).

**Table 2:** The incidence of ICIs-related respiratory disorders in different treatment regimens.

ICIs-related respiratory disorders	ICIs + Chemotherapy VS. Chemotherapy n/n (%)		ICIs VS. Chemotherapy n/n (%)		Double ICIs VS. single ICI n/n (%)		Total n/n (%)
	ICIs + Chemotherapy	Chemotherapy	ICIs	Chemotherapy	Double ICIs	single ICI	
Chronic obstructive pulmonary disease	26/1969 (1.32%)	16/1411 (1.13%)	35/3118 (1.12%)	20/2627 (0.76%)	5/560 (0.89%)	11/560 (1.96%)	113/10245 (1.10%)
Dyspnea	23/2235 (1.03%)	18/1542 (1.17%)	54/3301 (1.64%)	35/2633 (1.33%)	25/684 (3.65%)	24/683 (3.51%)	179/11078 (1.62%)
Hemoptysis	21/2235 (0.94%)	12/1542 (0.78%)	22/3455 (0.64%)	18/2783 (0.65%)	2/560 (0.36%)	2/560 (0.36%)	77/11135 (0.69%)
Interstitial lung disease	7/1573 (0.45%)	4/1082 (0.37%)	18/2622 (0.69%)	3/2005 (0.15%)	2/560 (0.36%)	2/560 (0.36%)	36/8402 (0.43%)
Pleural effusion	28/2235 (1.25%)	12/1542 (0.78%)	58/3455 (1.68%)	23/2783 (0.83%)	9/684 (1.32%)	8/683 (1.17%)	138/11382 (1.21%)
Pneumonitis	56/2275 (2.46%)	20/1580 (1.27%)	98/3455 (2.84%)	6/2783 (0.22%)	44/684 (6.43%)	21/683 (3.07%)	245/11460 (2.14%)
Pneumothorax	6/2235 (0.27%)	7/1542 (0.45%)	11/3168 (0.35%)	9/2515 (0.36%)	2/406 (0.49%)	2/404 (0.50%)	37/10270 (0.36%)
Pulmonary embolism	31/1969 (1.57%)	17/1411 (1.20%)	64/3455 (1.85%)	35/2783 (1.26%)	5/560 (0.89%)	10/560 (1.79%)	162/10738 (1.51%)
Pulmonary hemorrhage	2/1029 (0.19%)	1/671 (0.15%)	9/2776 (0.32%)	10/2155 (0.46%)	3/684 (0.44%)	6/683 (0.88%)	31/7998 (0.39%)
Respiratory failure	16/2235 (0.72%)	9/1559 (0.58%)	32/3455 (0.93%)	23/2783 (0.83%)	7/684 (1.02%)	6/683 (0.88%)	93/11399 (0.82%)

### Risk of chronic obstructive pulmonary disease

**Figure 4:**
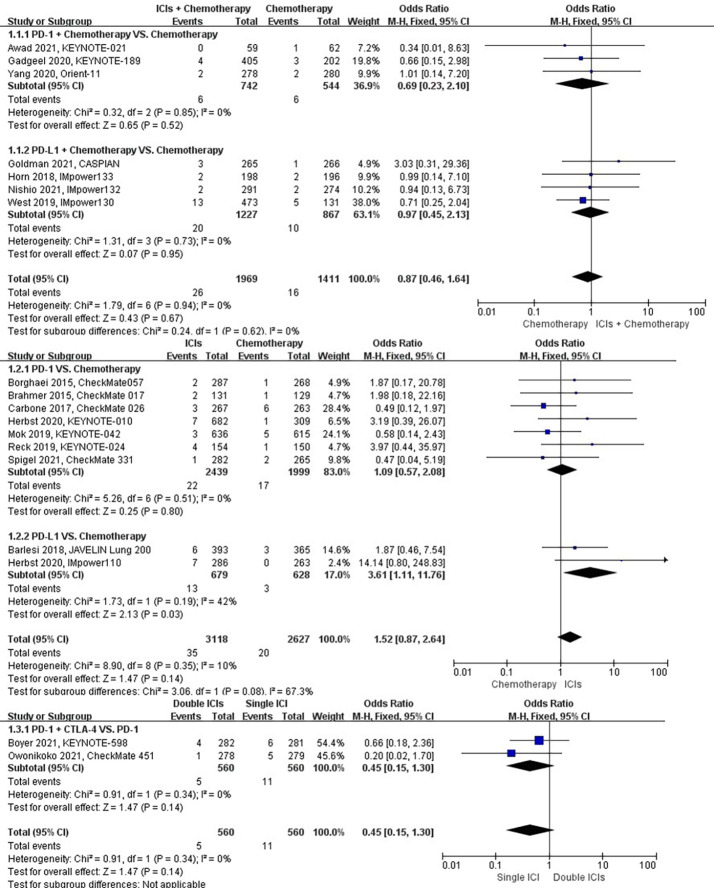
The forest plot of different treatment regimens on chronic obstructive pulmonary disease. Subgroup analyses investigated ICIs plus chemotherapy vs. chemotherapy, ICIs vs. chemotherapy and double ICIs vs. single ICI. CI, confidence interval.

The different treatment regimens on the risk of chronic obstructive pulmonary disease were presented for 18 datasets (ICIs plus chemotherapy [n = 1969] vs. chemotherapy [n = 1411]; ICIs [n = 3118] vs. chemotherapy [n = 2627]; double ICIs [n = 560] vs. single ICI [n = 560]). Compared with chemotherapy, ICIs plus chemotherapy (*P* = 0.67; SMD: 0.87; 95% CI: 0.46, 1.64) or ICIs (*P* = 0.14; SMD: 1.52; 95% CI: 0.87, 2.64) did not significantly change the incidence of chronic obstructive pulmonary disease with low evidence of heterogeneity among the studies (*I^2^* = 0% and 10%). Double ICIs (*P* = 0.34; SMD: 0.45; 95% CI: 0.15, 1.30) did not significantly change the incidence of chronic obstructive pulmonary disease when compared with single ICI with low evidence of heterogeneity among the studies (*I^2^* = 0%) (**Figure 4**).

### Risk of dyspnea

**Figure 5:**
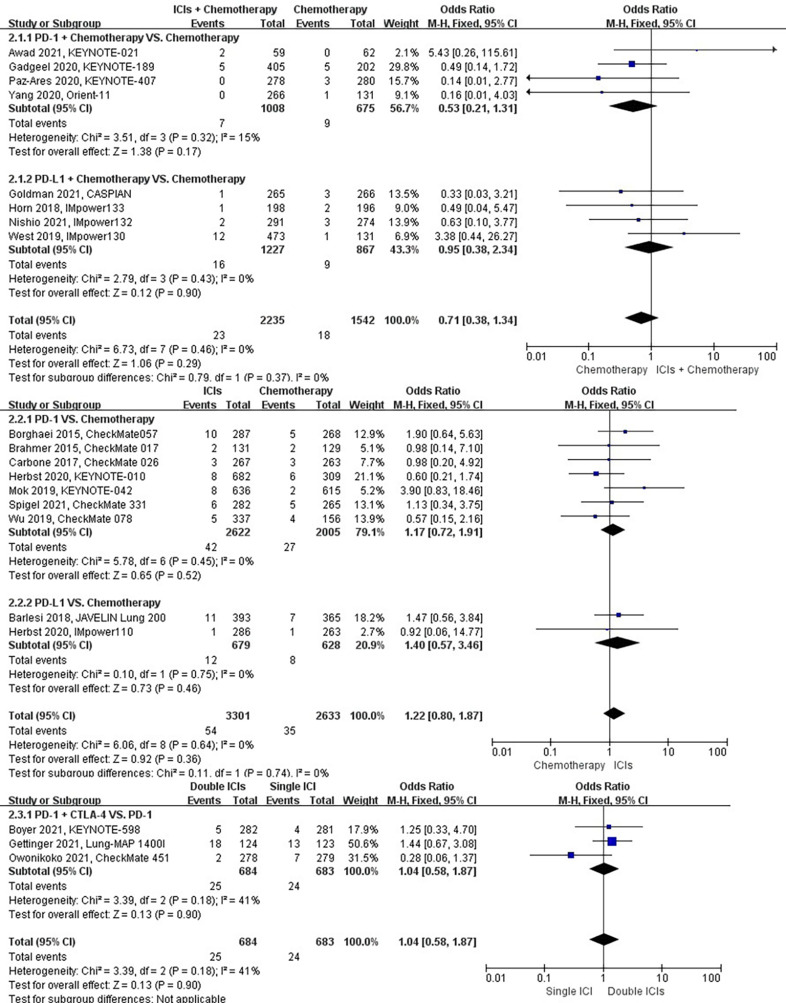
The forest plot of different treatment regimens on dyspnea. Subgroup analyses investigated ICIs plus chemotherapy vs. chemotherapy, ICIs vs. chemotherapy and double ICIs vs. single ICI. CI, confidence interval.

The different treatment regimens on the risk of dyspnea were presented for 19 datasets (ICIs plus chemotherapy [n = 2235] vs. chemotherapy [n = 1542]; ICIs [n = 3301] vs. chemotherapy [n = 2633]; double ICIs [n = 684] vs. single ICI [n = 683]). Compared with chemotherapy, ICIs plus chemotherapy (*P* = 0.29; SMD: 0.71; 95% CI: 0.38, 1.34) or ICIs (*P* = 0.36; SMD: 1.22; 95% CI: 0.80, 1.87) did not significantly change the incidence of dyspnea with low evidence of heterogeneity among the studies (*I^2^* = 0% and 0%). Double ICIs (*P* = 0.90; SMD: 1.04; 95% CI: 0.58, 1.87) did not significantly change the incidence of dyspnea when compared with single ICI with moderate evidence of heterogeneity among the studies (*I^2^* = 41%) (**Figure 5**).

### Risk of hemoptysis

**Figure 6:**
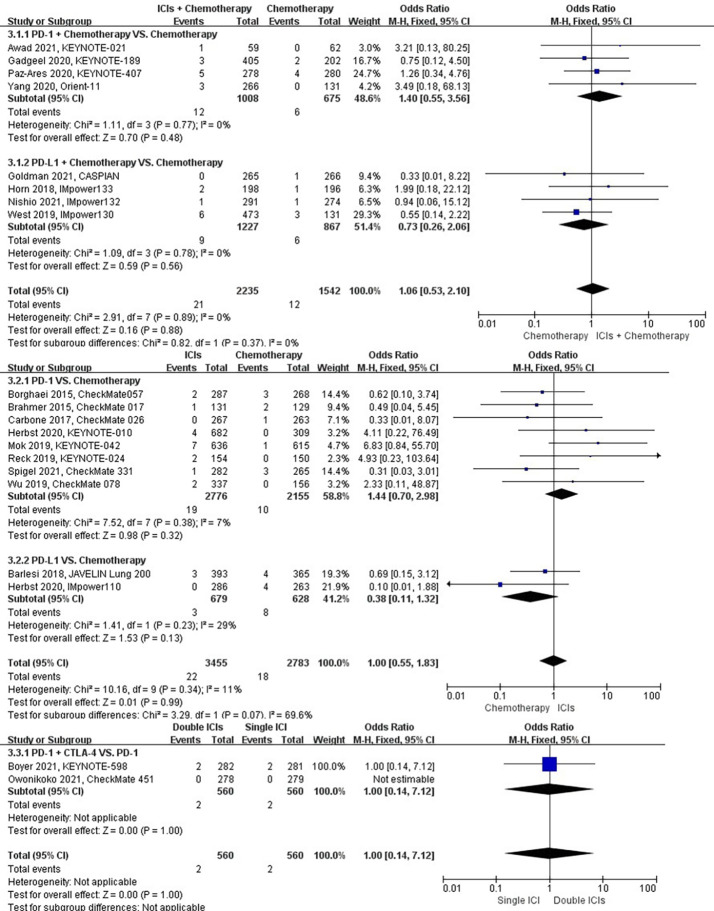
The forest plot of different treatment regimens on hemoptysis. Subgroup analyses investigated ICIs plus chemotherapy vs. chemotherapy, ICIs vs. chemotherapy and double ICIs vs. single ICI. CI, confidence interval.

The different treatment regimens on the risk of hemoptysis were presented for 20 datasets (ICIs plus chemotherapy [n = 2235] vs. chemotherapy [n = 1542]; ICIs [n = 3455] vs. chemotherapy [n = 2783]; double ICIs [n = 560] vs. single ICI [n = 560]). Compared with chemotherapy, ICIs plus chemotherapy (*P* = 0.88; SMD: 1.06; 95% CI: 0.53, 2.10) or ICIs (*P* = 0.99; SMD: 1.00; 95% CI: 0.55, 1.83) did not significantly change the incidence of hemoptysis with low evidence of heterogeneity among the studies (*I^2^* = 0% and 11%). Double ICIs (*P* = 1.00; SMD: 1.00; 95% CI: 0.14, 7.12) did not significantly change the incidence of hemoptysis compared with single ICI (**Figure 6**).

### Risk of interstitial lung disease

**Figure 7:**
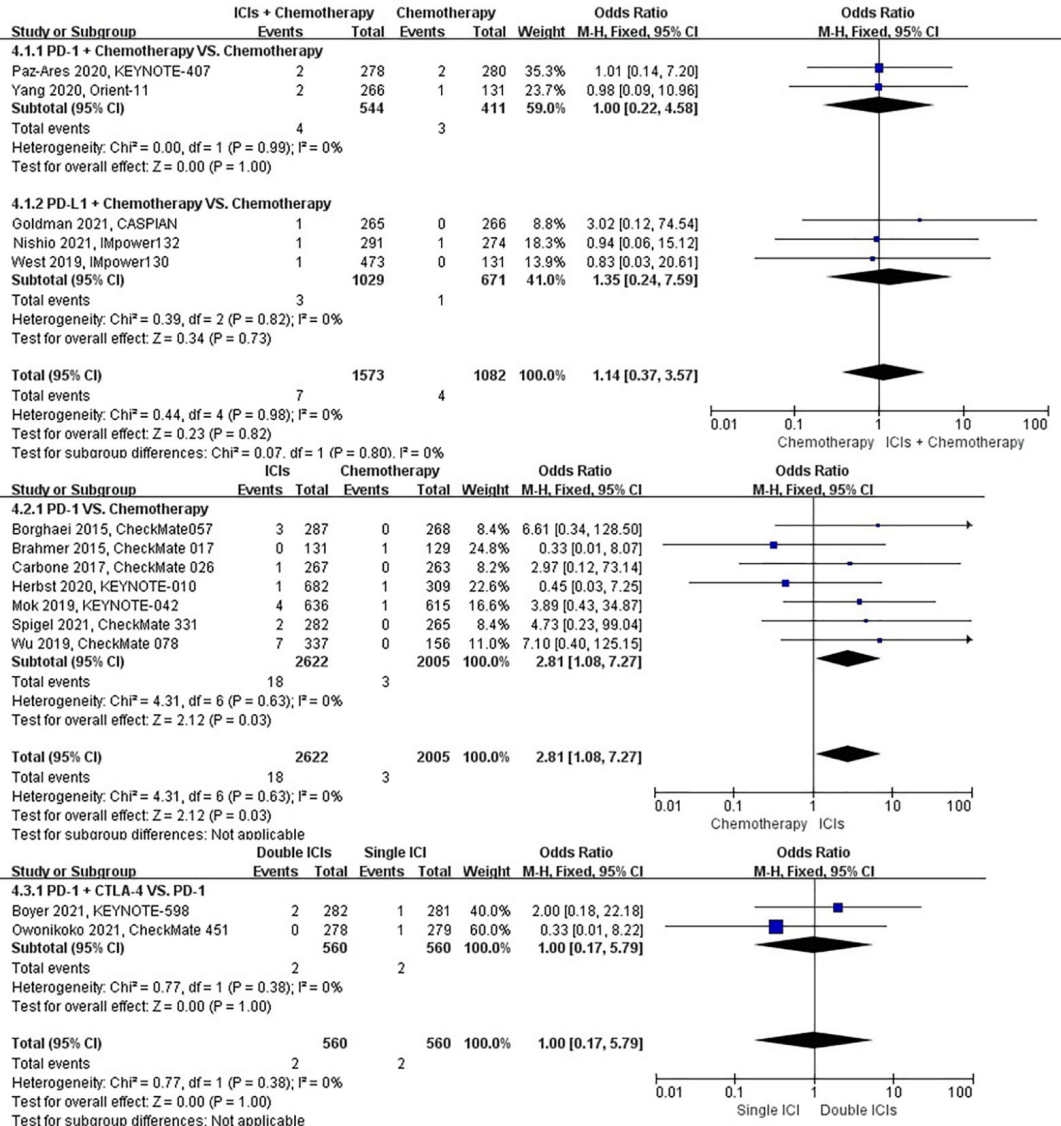
The forest plot of different treatment regimens on interstitial lung disease. Subgroup analyses investigated ICIs plus chemotherapy vs. chemotherapy, ICIs vs. chemotherapy and double ICIs vs. single ICI. CI, confidence interval.

The different treatment regimens on the risk of interstitial lung disease were presented for 15 datasets (ICIs plus chemotherapy [n = 1573] vs. chemotherapy [n = 1082]; ICIs [n = 2622] vs. chemotherapy [n = 2005]; double ICIs [n = 560] vs. single ICI [n = 560]). Compared with chemotherapy, ICIs plus chemotherapy (*P* = 0.82; SMD: 1.14; 95% CI: 0.37, 3.57) did not significantly change the incidence of interstitial lung disease with low evidence of heterogeneity among the studies (*I^2^* = 0%). ICIs (*P* = 0.03; SMD: 2.81; 95% CI: 1.08, 7.27) significantly increased the risk of suffering interstitial lung disease with low evidence of heterogeneity among the studies (*I^2^* = 0%). Double ICIs (*P* = 1.00; SMD: 1.00; 95% CI: 0.17, 5.79) did not significantly change the incidence of interstitial lung disease when compared with single ICI with low evidence of heterogeneity among the studies (*I^2^* = 0%) (**Figure 7**).

### Risk of pleural effusion

**Figure 8:**
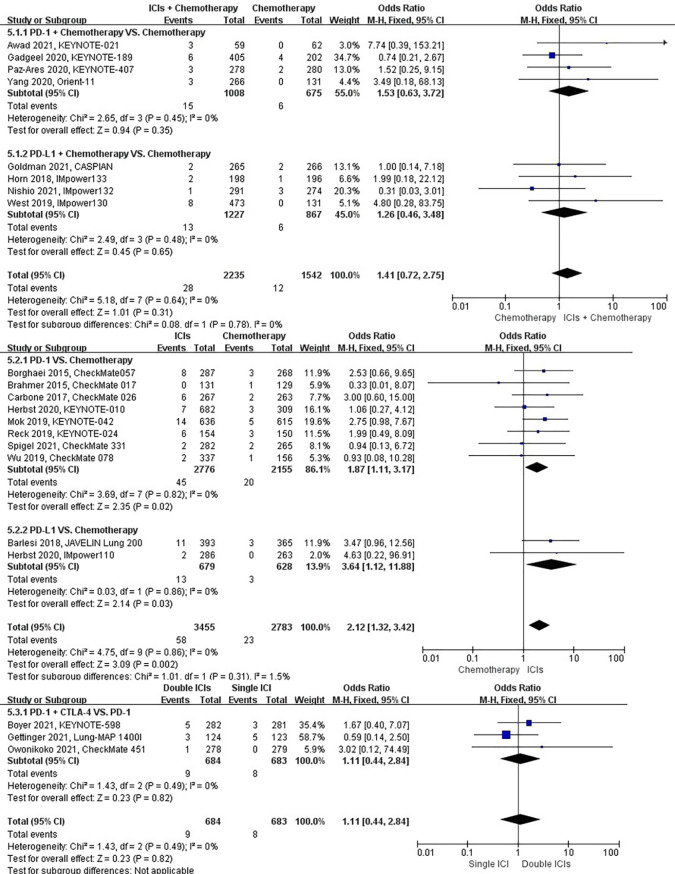
The forest plot of different treatment regimens on pleural effusion. Subgroup analyses investigated ICIs plus chemotherapy vs. chemotherapy, ICIs vs. chemotherapy and double ICIs vs. single ICI. CI, confidence interval.

The different treatment regimens on the risk of pleural effusion were presented for 20 datasets (ICIs plus chemotherapy [n = 2235] vs. chemotherapy [n = 2542]; ICIs [n = 3455] vs. chemotherapy [n = 2783]; double ICIs [n = 684] vs. single ICI [n = 683]). Compared with chemotherapy, ICIs plus chemotherapy (*P* = 0.31; SMD: 1.41; 95% CI: 0.72, 2.75) did not significantly change the incidence of pleural effusion with low evidence of heterogeneity among the studies (*I^2^* = 0%). ICIs (*P* = 0.002; SMD: 2.12; 95% CI: 1.32, 3.42) significantly increased the risk of suffering pleural effusion with low evidence of heterogeneity among the studies (*I^2^* = 0%). Double ICIs (*P* = 0.82; SMD: 1.11; 95% CI: 0.44, 2.84) did not significantly change the incidence of pleural effusion when compared with single ICI with low evidence of heterogeneity among the studies (*I^2^* = 0%) (**Figure 8**).

### Risk of pneumonitis

**Figure 9:**
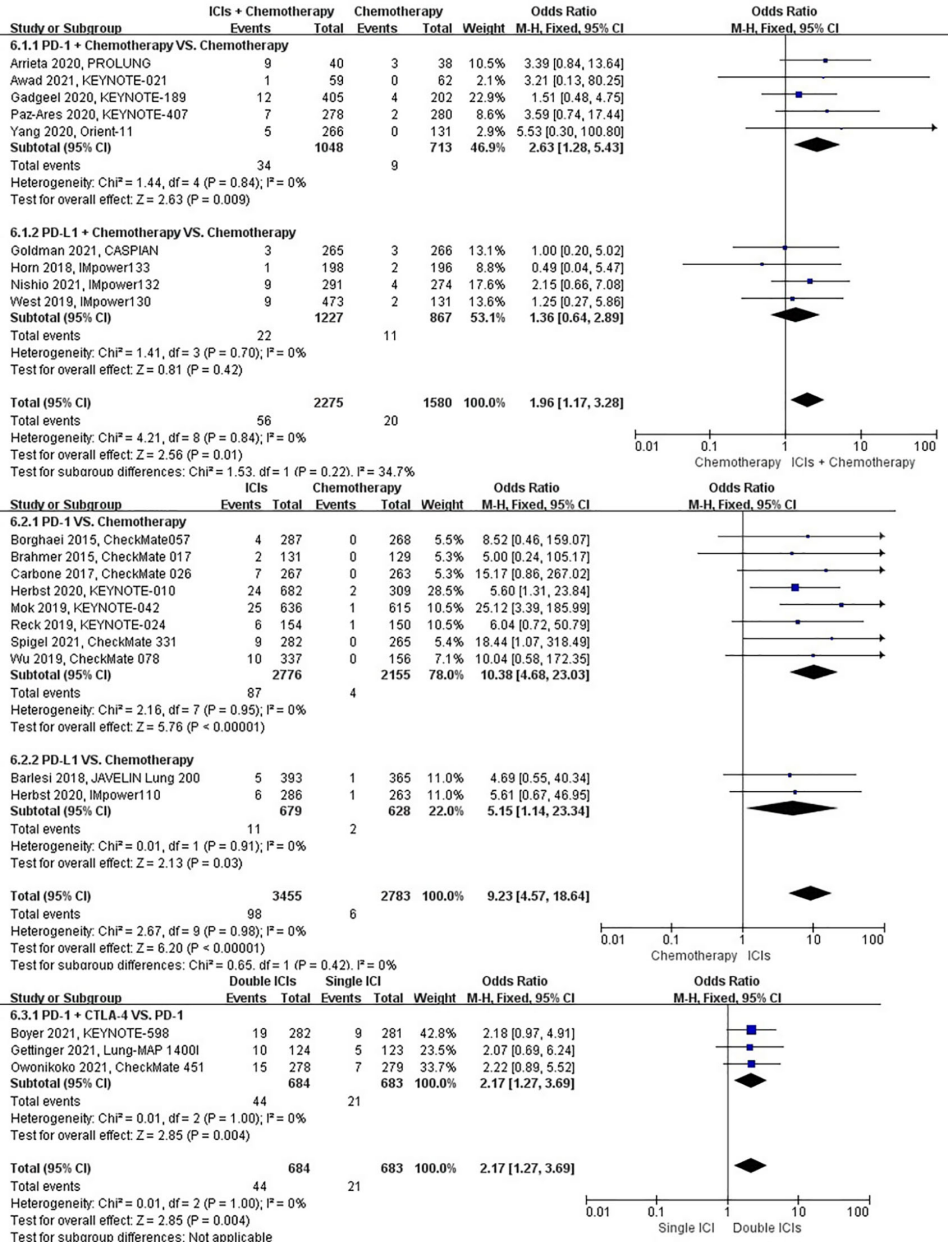
The forest plot of different treatment regimens on pneumonitis. Subgroup analyses investigated ICIs plus chemotherapy vs. chemotherapy, ICIs vs. chemotherapy and double ICIs vs. single ICI. CI, confidence interval.

The different treatment regimens on the risk of pneumonitis were presented for 22 datasets (ICIs plus chemotherapy [n = 2275] vs. chemotherapy [n = 1580]; ICIs [n = 3455] vs. chemotherapy [n = 2783]; double ICIs [n = 684] vs. single ICI [n = 683]). Compared with chemotherapy, ICIs plus chemotherapy (*P* = 0.01; SMD: 1.96; 95% CI: 1.17, 3.28) and ICIs (*P* < 0.00001; SMD: 9.23; 95% CI: 4.57, 18.64) significantly increased the risk of suffering pneumonitis with low evidence of heterogeneity among the studies (*I^2^* = 0% and 0%). Double ICIs (*P* = 0.004; SMD: 2.17; 95% CI: 1.27, 3.69) significantly increased the risk of suffering pneumonitis with low evidence of heterogeneity among the studies (*I^2^* = 0%) (**Figure 9**).

### Risk of pneumothorax

**Figure 10:**
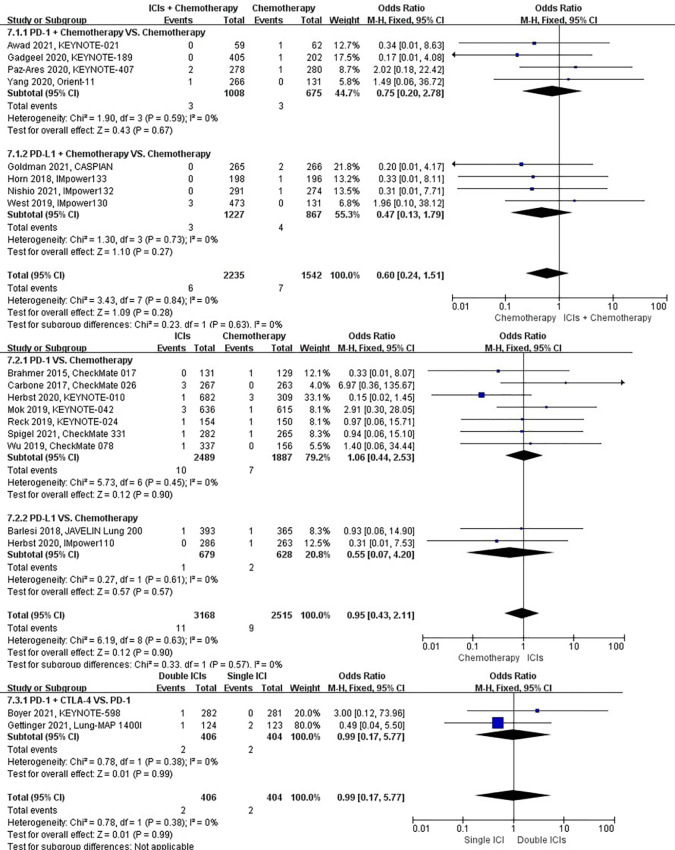
The forest plot of different treatment regimens on pneumothorax. Subgroup analyses investigated ICIs plus chemotherapy vs. chemotherapy, ICIs vs. chemotherapy and double ICIs vs. single ICI. CI, confidence interval.

The different treatment regimens on the risk of pneumothorax were presented for 19 datasets (ICIs plus chemotherapy [n = 2235] vs. chemotherapy [n = 1542]; ICIs [n = 3168] vs. chemotherapy [n = 2515]; double ICIs [n = 406] vs. single ICI [n = 404]). Compared with chemotherapy, ICIs plus chemotherapy (*P* = 0.28; SMD: 0.60; 95% CI: 0.24, 1.51) or ICIs (*P* = 0.90; SMD: 0.95; 95% CI: 0.43, 2.11) did not significantly change the incidence of pneumothorax with low evidence of heterogeneity among the studies (*I^2^* = 0% and 0%). Double ICIs (*P* = 0.99; SMD: 0.99; 95% CI: 0.17, 5.77) did not significantly change the incidence of pneumothorax when compared with single ICI with low evidence of heterogeneity among the studies (*I^2^* = 0%) (**Figure 10**).

### Risk of pulmonary embolism

**Figure 11:**
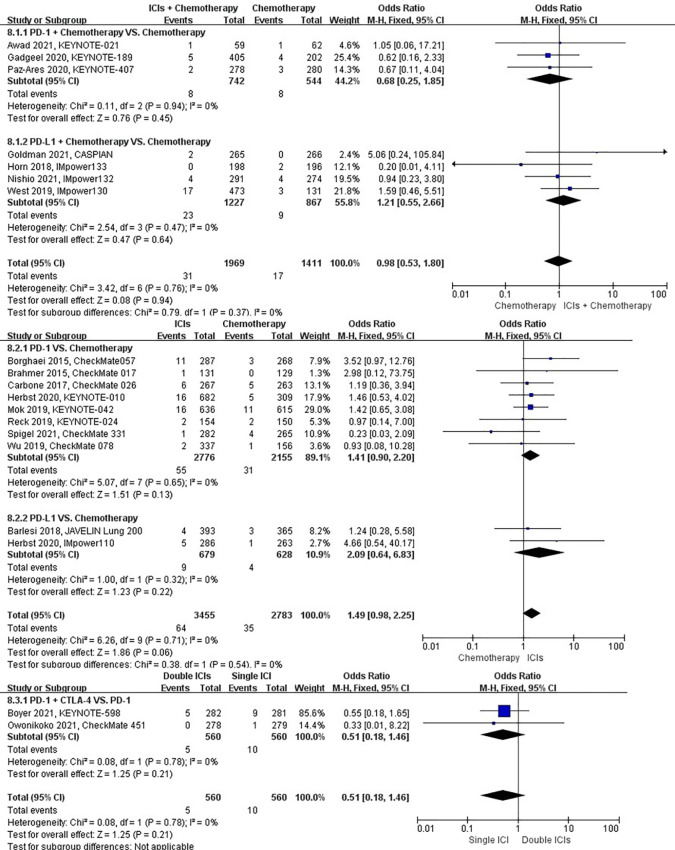
The forest plot of different treatment regimens on pulmonary embolism. Subgroup analyses investigated ICIs plus chemotherapy vs. chemotherapy, ICIs vs. chemotherapy and double ICIs vs. single ICI. CI, confidence interval.

The different treatment regimens on the risk of pulmonary embolism were presented for 19 datasets (ICIs plus chemotherapy [n = 1969] vs. chemotherapy [n = 1411]; ICIs [n = 3455] vs. chemotherapy [n = 2783]; double ICIs [n = 560] vs. single ICI [n = 560]). Compared with chemotherapy, ICIs plus chemotherapy (*P* = 0.94; SMD: 0.98; 95% CI: 0.53, 1.80) or ICIs (*P* = 0.06; SMD: 1.49; 95% CI: 0.98, 2.25) did not significantly change the incidence of pulmonary embolism with low evidence of heterogeneity among the studies (*I^2^* = 0% and 0%). Double ICIs (*P* = 0.21; SMD: 0.51; 95% CI: 0.18, 1.46) did not significantly change the incidence of pulmonary embolism when compared with single ICI with low evidence of heterogeneity among the studies (*I^2^* = 0%) (**Figure 11**).

### Risk of pulmonary hemorrhage

**Figure 12:**
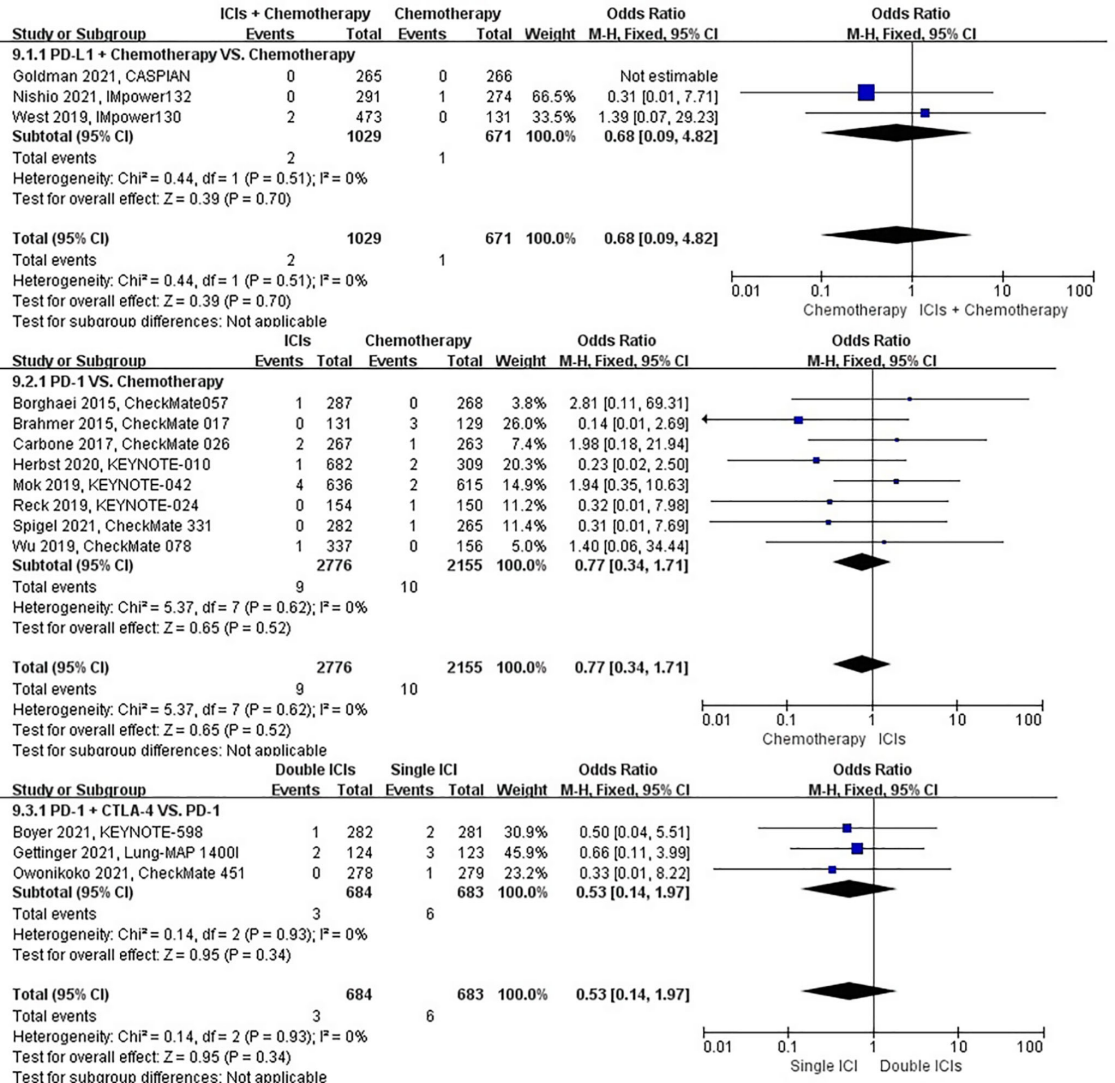
The forest plot of different treatment regimens on pulmonary hemorrhage. Subgroup analyses investigated ICIs plus chemotherapy vs. chemotherapy, ICIs vs. chemotherapy and double ICIs vs. single ICI. CI, confidence interval.

The different treatment regimens on the risk of pulmonary hemorrhage were presented for 15 datasets (ICIs plus chemotherapy [n = 1029] vs. chemotherapy [n = 671]; ICIs [n = 2776] vs. chemotherapy [n = 2155]; double ICIs [n = 684] vs. single ICI [n = 683]). Compared with chemotherapy, ICIs plus chemotherapy (*P* = 0.70; SMD: 0.68; 95% CI: 0.09, 4.82) or ICIs (*P* = 0.52; SMD: 0.77; 95% CI: 0.34, 1.71) did not significantly change the incidence of pulmonary hemorrhage with low evidence of heterogeneity among the studies (*I^2^* = 0% and 0%). Double ICIs (*P* = 0.34; SMD: 0.53; 95% CI: 0.14, 1.97) did not significantly change the incidence of pulmonary hemorrhage when compared with single ICI with low evidence of heterogeneity among the studies (*I^2^* = 0%) (**Figure 12**).

### Risk of respiratory failure

**Figure 13:**
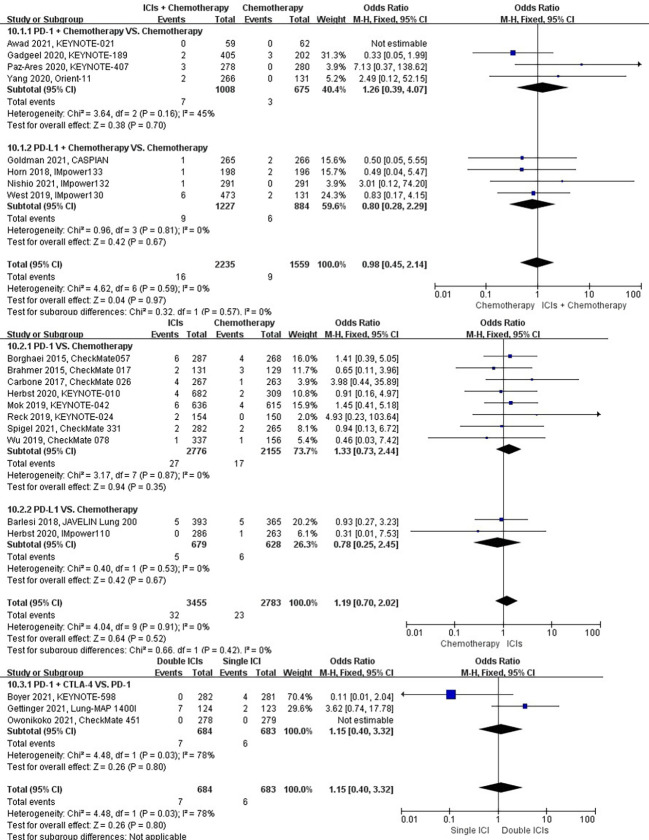
The forest plot of different treatment regimens on respiratory failure. Subgroup analyses investigated ICIs plus chemotherapy vs. chemotherapy, ICIs vs. chemotherapy and double ICIs vs. single ICI. CI, confidence interval.

The different treatment regimens on the risk of respiratory failure were presented for 20 datasets (ICIs plus chemotherapy [n = 2235] vs. chemotherapy [n = 1559]; ICIs [n = 3455] vs. chemotherapy [n = 2783]; double ICIs [n = 684] vs. single ICI [n = 683]). Compared with chemotherapy, ICIs plus chemotherapy (*P* = 0.97; SMD: 0.98; 95% CI: 0.45, 2.14) or ICIs (*P* = 0.52; SMD: 1.19; 95% CI: 0.70, 2.02) did not significantly change the incidence of respiratory failure with low evidence of heterogeneity among the studies (*I^2^* = 0% and 0%). Double ICIs (*P* = 0.80; SMD: 1.15; 95% CI: 0.40, 3.32) did not significantly change the incidence of respiratory failure when compared with single ICI with high evidence of heterogeneity among the studies (I^2^ = 78%) (**Figure 13**).

## Discussion

As the first FDA-approved ICI on anti-cancer, ipilimumab was used to treat advanced melanoma in 2011 (56). In the next decade, the use of ICIs in cancer treatment rapidly increased, including numerous breakthroughs, expanded treatment landscape for many malignancies, and improved outcomes, specifically NSCLC (57–59). Nivolumab, a PD-1 inhibitor, brought a promising outcome when it was effectively used as a second-line therapy for patients with advanced NSCLC. In the next phase III trials on advanced squamous and non-squamous NSCLC, nivolumab achieved inspiring results of OS and objective response rate (ORR) (37, 39). In recent years, several phase III clinical trials demonstrated a superior improvement in OS and more durable responses with ICIs or ICIs plus chemotherapy than chemotherapy (27, 29, 30, 32, 33, 44, 60, 61). The clinical choice mainly depends on disease burden, PD-L1 expression, and tumor mutation profile of the tumor. In comparison, the growth of ICIs in SCLC could be more satisfactory. ICIs merely had an achievement that PD-L1 inhibitors plus platinum-based chemotherapy conducted as first-line treatment of extensive-stage SCLC (46, 62).

Based on the extremely rapid growth of ICIs, various irAEs were reported. In general, irAEs are usual due to nonspecific immunostimulation, leading to autoimmunity, tissue damage, and organ-specific inflammation (63). They can be divided into cytokine release syndrome and cardiac, pulmonary, dermatologic, endocrine, neurologic, ocular, renal, rheumatologic, gastrointestinal, and hepatic toxicities (64, 65). Dermatologic, endocrine, gastrointestinal, and hepatic toxicities are shared among ICI-treated patients (65, 66). Cardiac and pulmonary toxicities are rare but potentially fatal (20, 67–70). In particular, pulmonary toxicity is rapidly progressive (71). Most of the irAEs caused by pulmonary toxicity occur during 10–12 weeks after ICI therapy, and the early symptoms are mild and nonspecific, such as cough (72). However, some ICI-treated patients may suffer severe ICIs-related respiratory disorders (SAE grade ≥ 3), such as chronic obstructive pulmonary disease, hemoptysis, interstitial lung disease, pleural effusion, pneumonitis, pulmonary embolism, and respiratory failure. Therefore, a broad range of diagnostic processes, including X-ray imaging, angiography, and laboratory analyses, are necessary for ICI-treated patients to distinguish pulmonary embolism, pleural effusion, pneumonitis, pneumothorax, and cancer progression.

In recent years, some researchers analyzed certain ICIs-related respiratory disorders in digestic and urologic cancer (73, 74). However, a study on comprehensive ICIs-related respiratory disorders in lung cancer is still warranted. In this study, we extracted 22 RCTs and analyzed the risk of top 10 most frequent ICIs-related respiratory disorders in patients with lung cancer. Overall, the analysis revealed that ICIs raise the risk of interstitial lung disease, pleural effusion, and pneumonitis compared with chemotherapy. Furthermore, ICIs plus chemotherapy brought a higher incidence of pneumonitis than chemotherapy. single ICI could provide a lower probability for patients to suffer pneumonitis than double ICIs. Also, other ICIs-related respiratory disorders, including chronic obstructive pulmonary disease, dyspnea, hemoptysis, pneumothorax, pulmonary embolism, pulmonary hemorrhage, and respiratory failure, were analyzed in this study. Still, no significant difference was observed among the different treatment regimens.

Previous studies have demonstrated that the incidence of ICIs-related interstitial lung disease ranges from 14.5% to 18.6% (75, 76). This study showed that ICIs were more likely to cause interstitial lung disease than chemotherapy. This is similar to other studies showing that ICIs cause interstitial lung disease more frequently than other drugs used to treat NSCLC, such as pemetrexed, erlotinib, gefitinib, docetaxel, gemcitabine, or crizotinib (77–83). However, the mechanisms regulating the occurrence of ICIs-related interstitial lung disease have not been fully elucidated so far. Elevated levels of inflammatory cytokines may be involved in the pathophysiology of irAEs (84, 85). The inflammatory cytokine interleukin 6 (IL-6) induces the differentiation of naive CD4 T cells into Th17 cells, which may be related to irAE occurrence (86). Th17 cells are critical mediators of various autoimmune diseases by producing IL-17 (87, 88). Likewise, tumor necrosis factor-α (TNF-α) has been associated with irAEs, and anti-TNF-α antibodies were found to improve severe irAEs (89). Patients with poorer performance score and cancer cachexia status have higher levels of inflammatory cytokines, such as IL-6 and TNF-α, and may be more prone to irAE-related diseases, such as ICIs-related interstitial lung disease (90, 91). PD-L1 inhibitors should be less toxic than PD1 inhibitors as they do not prevent the interaction between PD-L2 and PD1 (92). Still, we could not confirm this difference in our study due to data limitations.

In this study, a total of 81 pleural effusion events occurred in the ICIs vs. chemotherapy group, among which 58 and 23 patients developed pleural effusion after applying ICIs and using chemotherapy, respectively; ICIs significantly increased the risk of pleural effusion compared with chemotherapy (*P* < 0.05). Pleural effusion has been reported as an irAE, but there are few studies on ICIs-related pleural effusion, most of which are case reports (93–95). Two patients were reported to develop recurrent pleural effusions that accumulated rapidly within days after each puncture and required multiple thoracentesis for the first 8 weeks after administration of nivolumab (95). In another study, a patient was not initially diagnosed with pleural dissemination or malignant pleural effusion. However, cytology and radiography or thoracoscopy did not find evidence of malignancy in the pleural effusion and malignant nodules, respectively. Hypoalbuminemia and cardiac insufficiency, which may cause pleural effusion, were also excluded. And the pleural effusion responded well after corticotherapy, suggesting that this may be an irAE (94). Pleural effusion is considered to be related to the pseudo-progression of the disease (95). However, so far, there is no detailed research to explain this. Moreover, the mechanism still needs to be elucidated, and further research is warranted.

In some RCTs, the incidence of ICIs-related pneumonitis was approximately 1.06% (95% CI: 0.53–2.11) for CTLA-4 inhibitors, 3.02% (95% CI: 2.31–3.93) for PD-1 inhibitors, and 7.09% (95% CI: 5.52–7.16) for PD-1 combined with CTLA-4 inhibitors (28, 30, 37, 39, 96–114). ICIs-related pneumonitis is hypothesized to be a chronic inflammatory state (114). Its symptoms are nonspecific and usually present with cough, dyspnea, shortness of breath, and hypoxia (115, 116). This study demonstrated that the risk of pneumonitis after treatment of PD-1 combined with CTLA-4 was higher than that of PD-1 alone, which is similar to the previous study (116) showing that the risks of pneumonitis (3.47-fold) and severe pneumonitis (3.48-fold) were higher with ipilimumab combined with nivolumab than nivolumab or ipilimumab alone. Therefore, the combination of CTLA-4 and PD-1 may cause a higher incidence of pneumonitis than either drug (110–112). CTLA-4 inhibitors attenuate T cell activation early in the immune response. PD-1 inhibitors can inhibit T cells later in peripheral tissue immune response. Therefore, we assumed that the combined application of PD-1 and CTLA-4 may be more prone to lung toxicity than either treatment alone; however, further studies are needed to reveal this molecular mechanism. In this study, we also found that ICIs with or without chemotherapy increased the risk of pneumonitis compared with chemotherapy alone. Interestingly, studies have demonstrated that patients with NSCLC treated with PD-L1 inhibitors have a higher incidence of pneumonitis than those with other cancer types. In a study that compared data from patients with lung cancer and other solid tumors, pneumonitis was more common in patients with lung cancer (26.4% vs. 10.3%) (117). Therefore, tumor damage to lung tissue may make the lungs more prone to side effects after treatment. With the increasing use of ICIs in more neoplastic diseases, the total burden of pneumonitis and mortality will undoubtedly increase.

This study has several limitations. First, NSCLC and SCLC were both investigated in the analysis. A retrospective study demonstrated that squamous cell cancer was a risk factor for ICIs-related pneumonitis (17). As a confounding factor, SCLC might increase heterogeneity to a certain degree. Second, there needed to be more datasets to build single-drug subgroups for certain drug analysis, such as avelumab and sintilimab. Third, the study did not involve LAG3, PD-L2, and other ICIs.

In conclusion, this study showed that ICI-based treatment, such as ICIs alone, ICIs plus chemotherapy, and double ICIs, can raise the incidences of some respiratory disorders in patients with lung cancer. It suggests that ICIs should be conducted based on a comprehensive consideration to prevent ICIs-related respiratory disorders. To a certain degree, this study might be provided to the clinician as a reference for ICI practice. Of course, more prospective and well-designed clinical trials, and larger sample size real-world studies on various ICIs are still needed to further evaluate therapeutic effects and ICIs-related adverse events.

## Data availability statement

The original contributions presented in the study are included in the article/**Supplementary Material**. Further inquiries can be directed to the corresponding authors.

## Author contributions

SW conducted the analysis, SL, JJ and LP collected and performed a preliminary analysis of references, HL designed the manuscript, SL and HL wrote the manuscript, LP, SW and LS revised the manuscript. All authors contributed to the article and approved the submitted version.

## Supplementary material

Supplementary Figure 1 The funnel plot for accessing publication bias: chronic obstructive pulmonary diseases.

Supplementary Figure 2 The funnel plot for accessing publication bias: dyspnea.

Supplementary Figure 3 The funnel plot for accessing publication bias: hemoptysis.

Supplementary Figure 4 The funnel plot for accessing publication bias: interstitial lung disease.

Supplementary Figure 5 The funnel plot for accessing publication bias: pleural effusion.

Supplementary Figure 6 The funnel plot for accessing publication bias: pneumonitis.

Supplementary Figure 7 The funnel plot for accessing publication bias: pneumothorax.

Supplementary Figure 8 The funnel plot for accessing publication bias: pulmonary embolism.

Supplementary Figure 9 The funnel plot for accessing publication bias: pulmonary hemorrhage.

Supplementary Figure 10 The funnel plot for accessing publication bias: respiratory failure.

## References

[1] Siegel RL Miller KD Fuchs HE Jemal A . Cancer statistics, 2022. CA Cancer J Clin (2022) 72(1):7–33. doi: 10.3322/caac.21708 35020204

[2] Howlader N Forjaz G Mooradian MJ Meza R Kong CY Cronin KA . The effect of advances in lung-cancer treatment on population mortality. N Engl J Med (2020) 383(7):640–9. doi: 10.1056/NEJMoa1916623 PMC8577315 32786189

[3] Amarnath S Mangus CW Wang JC Wei F He A Kapoor V . The PDL1-PD1 axis converts human TH1 cells into regulatory T cells. Sci Transl Med (2011) 3(111):111ra20. doi: 10.1126/scitranslmed.3003130 PMC3235958 22133721

[4] Francisco LM Salinas VH Brown KE Vanguri VK Freeman GJ Kuchroo VK . PD-L1 regulates the development, maintenance, and function of induced regulatory T cells. J Exp Med (2009) 206(13):3015–29. doi: 10.1084/jem.20090847 PMC2806460 20008522

[5] Kaen DL Minatta N Russo A Malapelle U de Miguel-Pérez D Rolfo C . Immunotherapy in lung cancer: Are the promises of long-term benefit finally met? Adv Exp Med Biol (2021) 1342:113–42. doi: 10.1007/978-3-030-79308-1_4 34972964

[6] Tivol EA Borriello F Schweitzer AN Lynch WP Bluestone JA Sharpe AH . Loss of CTLA-4 leads to massive lymphoproliferation and fatal multiorgan tissue destruction, revealing a critical negative regulatory role of CTLA-4. Immunity (1995) 3(5):541–7. doi: 10.1016/1074-7613(95)90125-6 7584144

[7] Chambers CA Sullivan TJ Allison JP . Lymphoproliferation in CTLA-4-deficient mice is mediated by costimulation-dependent activation of CD4+ T cells. Immunity (1997) 7(6):885–95. doi: 10.1016/S1074-7613(00)80406-9 9430233

[8] Waterhouse P Penninger JM Timms E Wakeham A Shahinian A Lee KP . Lymphoproliferative disorders with early lethality in mice deficient in ctla-4. Science (1995) 270(5238):985–8. doi: 10.1126/science.270.5238.985 7481803

[9] Walker LS Sansom DM . The emerging role of CTLA4 as a cell-extrinsic regulator of T cell responses. Nat Rev Immunol (2011) 11(12):852–63. doi: 10.1038/nri3108 22116087

[10] Kisielow M Kisielow J Capoferri-Sollami G Karjalainen K . Expression of lymphocyte activation gene 3 (LAG-3) on b cells is induced by T cells. Eur J Immunol (2005) 35(7):2081–8. doi: 10.1002/eji.200526090 15971272

[11] Wang Y Hou K Jin Y Bao B Tang S Qi J . Lung adenocarcinoma-specific three-integrin signature contributes to poor outcomes by metastasis and immune escape pathways. J Transl Int Med (2021) 31;9(4):249–63. doi: 10.2478/jtim-2021-0046 PMC8802404 35136724

[12] Giroux Leprieur E Dumenil C Julie C Giraud V Dumoulin J Labrune S . Immunotherapy revolutionises non-small-cell lung cancer therapy: Results, perspectives and new challenges. Eur J Cancer (2017) 78:16–23. doi: 10.1016/j.ejca.2016.12.041 28407528

[13] Naidoo J Page DB Li BT Connell LC Schindler K Lacouture ME . Toxicities of the anti-PD-1 and anti-PD-L1 immune checkpoint antibodies. Ann Oncol (2015) 26(12):2375–91. doi: 10.1093/annonc/mdv383 PMC6267867 26371282

[14] Champiat S Lambotte O Barreau E Belkhir R Berdelou A Carbonnel F . Management of immune checkpoint blockade dysimmune toxicities: a collaborative position paper. Ann Oncol (2016) 27(4):559–74. doi: 10.1093/annonc/mdv623 26715621

[15] Bishay K Tandon P Bourassa-Blanchette S Laurie SA McCurdy JD . The risk of diarrhea and colitis in patients with lung cancer treated with immune checkpoint inhibitors: a systematic review and meta-analysis. Curr Oncol (2020) 27(5):e486–e94. doi: 10.3747/co.27.6251 PMC7606037 33173388

[16] Huo G Liu W Chen P . Inhibitors of PD-1 in non-small cell lung cancer: A meta-analysis of clinical and molecular features. Front Immunol (2022) 13:875093. doi: 10.3389/fimmu.2022.875093 35479081 PMC9037098

[17] Zhou P Zhao X Wang G . Risk factors for immune checkpoint inhibitor-related pneumonitis in cancer patients: A systemic review and meta-analysis. Respiration (2022) 101(11):1035–50. doi: 10.1159/000526141 36108598

[18] Kou L Wen Q Xie X Chen X Li J Li Y . Adverse events of immune checkpoint inhibitors for patients with digestive system cancers: A systematic review and meta-analysis. Front Immunol (2022) 13:1013186. doi: 10.3389/fimmu.2022.1013186 36341450 PMC9634077

[19] Liu S Gao W Ning Y Zou X Zhang W Zeng L . Cardiovascular toxicity with PD-1/PD-L1 inhibitors in cancer patients: A systematic review and meta-analysis. Front Immunol (2022) 13:908173. doi: 10.3389/fimmu.2022.908173 35880172 PMC9307961

[20] Wang DY Salem JE Cohen JV Chandra S Menzer C Ye F . Fatal toxic effects associated with immune checkpoint inhibitors: A systematic review and meta-analysis. JAMA Oncol (2018) 4(12):1721–8. doi: 10.1001/jamaoncol.2018.3923 PMC6440712 30242316

[21] Page MJ McKenzie JE Bossuyt PM Boutron I Hoffmann TC Mulrow CD . The PRISMA 2020 statement: an updated guideline for reporting systematic reviews. Syst Rev (2021) 10(1):89. doi: 10.1186/s13643-021-01626-4 33781348 PMC8008539

[22] Moher D Liberati A Tetzlaff J Altman DG . Preferred reporting items for systematic reviews and meta-analyses: the PRISMA statement. Int J Surg (2010) 8(5):336–41. doi: 10.1016/j.ijsu.2010.02.007 20171303

[23] Higgins JP Altman DG Gøtzsche PC Jüni P Moher D Oxman AD . The cochrane collaboration's tool for assessing risk of bias in randomised trials. Bmj (2011) 343:d5928. doi: 10.1136/bmj.d5928 22008217 PMC3196245

[24] Irwig L Macaskill P Berry G Glasziou P . Bias in meta-analysis detected by a simple, graphical test. Graphical test is itself biased Bmj (1998) 316(7129):470. PMC2665595 9492687

[25] DerSimonian R Laird N . Meta-analysis in clinical trials. Control Clin Trials (1986) 7(3):177–88. doi: 10.1016/0197-2456(86)90046-2 3802833

[26] Higgins JP Thompson SG Deeks JJ Altman DG . Measuring inconsistency in meta-analyses. BMJ (2003) 327(7414):557–60. doi: 10.1136/bmj.327.7414.557 PMC192859 12958120

[27] Gandhi L Rodríguez-Abreu D Gadgeel S Esteban E Felip E De Angelis F . Pembrolizumab plus chemotherapy in metastatic non-Small-Cell lung cancer. N Engl J Med (2018) 378(22):2078–92. doi: 10.1056/NEJMoa1801005 29658856

[28] Herbst RS Baas P Kim DW Felip E Pérez-Gracia JL Han JY . Pembrolizumab versus docetaxel for previously treated, PD-L1-positive, advanced non-small-cell lung cancer (KEYNOTE-010): a randomised controlled trial. Lancet (2016) 387(10027):1540–50. doi: 10.1016/S0140-6736(15)01281-7 26712084

[29] Paz-Ares L Luft A Vicente D Tafreshi A Gümüş M Mazières J . Pembrolizumab plus chemotherapy for squamous non-Small-Cell lung cancer. N Engl J Med (2018) 379(21):2040–51. doi: 10.1056/NEJMoa1810865 30280635

[30] Reck M Rodríguez-Abreu D Robinson AG Hui R Csőszi T Fülöp A . Pembrolizumab versus chemotherapy for PD-L1-Positive non-Small-Cell lung cancer. N Engl J Med (2016) 375(19):1823–33. doi: 10.1056/NEJMoa1606774 27718847

[31] Felip E Altorki N Zhou C Csőszi T Vynnychenko I Goloborodko O . Adjuvant atezolizumab after adjuvant chemotherapy in resected stage IB-IIIA non-small-cell lung cancer (IMpower010): a randomised, multicentre, open-label, phase 3 trial. Lancet (2021) 398(10308):1344–57. doi: 10.1016/S0140-6736(21)02098-5 34555333

[32] Hellmann MD Paz-Ares L Bernabe Caro R Zurawski B Kim SW Carcereny Costa E . Nivolumab plus ipilimumab in advanced non-Small-Cell lung cancer. N Engl J Med (2019) 381(21):2020–31. doi: 10.1056/NEJMoa1910231 31562796

[33] Sezer A Kilickap S Gümüş M Bondarenko I Özgüroğlu M Gogishvili M . Cemiplimab monotherapy for first-line treatment of advanced non-small-cell lung cancer with PD-L1 of at least 50%: a multicentre, open-label, global, phase 3, randomised, controlled trial. Lancet (2021) 397(10274):592–604. doi: 10.1016/S0140-6736(21)00228-2 33581821

[34] Arrieta O Barrón F Ramírez-Tirado LA Zatarain-Barrón ZL Cardona AF Díaz-García D . Efficacy and safety of pembrolizumab plus docetaxel vs docetaxel alone in patients with previously treated advanced non-small cell lung cancer: The PROLUNG phase 2 randomized clinical trial. JAMA Oncol (2020) 6(6):856–64. doi: 10.1001/jamaoncol.2020.0409 PMC7290397 32271354

[35] Awad MM Gadgeel SM Borghaei H Patnaik A Yang JC Powell SF . Long-term overall survival from KEYNOTE-021 cohort G: Pemetrexed and carboplatin with or without pembrolizumab as first-line therapy for advanced nonsquamous NSCLC. J Thorac Oncol (2021) 16(1):162–8. doi: 10.1016/j.jtho.2020.09.015 33069888

[36] Barlesi F Vansteenkiste J Spigel D Ishii H Garassino M de Marinis F . Avelumab versus docetaxel in patients with platinum-treated advanced non-small-cell lung cancer (JAVELIN lung 200): an open-label, randomised, phase 3 study. Lancet Oncol (2018) 19(11):1468–79. doi: 10.1016/S1470-2045(18)30673-9 30262187

[37] Borghaei H Paz-Ares L Horn L Spigel DR Steins M Ready NE . Nivolumab versus docetaxel in advanced nonsquamous non-Small-Cell lung cancer. N Engl J Med (2015) 373(17):1627–39. doi: 10.1056/NEJMoa1507643 PMC5705936 26412456

[38] Boyer M Şendur MAN Rodríguez-Abreu D Park K Lee DH Çiçin I . Pembrolizumab plus ipilimumab or placebo for metastatic non-Small-Cell lung cancer with PD-L1 tumor proportion score ≥ 50%: Randomized, double-blind phase III KEYNOTE-598 study. J Clin Oncol (2021) 39(21):2327–38. doi: 10.1200/JCO.20.03579 33513313

[39] Brahmer J Reckamp KL Baas P Crinò L Eberhardt WE Poddubskaya E . Nivolumab versus docetaxel in advanced squamous-cell non-Small-Cell lung cancer. N Engl J Med (2015) 373(2):123–35. doi: 10.1056/NEJMoa1504627 PMC4681400 26028407

[40] Carbone DP Reck M Paz-Ares L Creelan B Horn L Steins M . First-line nivolumab in stage IV or recurrent non-Small-Cell lung cancer. N Engl J Med (2017) 376(25):2415–26. doi: 10.1056/NEJMoa1613493 PMC6487310 28636851

[41] Gadgeel S Rodríguez-Abreu D Speranza G Esteban E Felip E Dómine M . Updated analysis from KEYNOTE-189: Pembrolizumab or placebo plus pemetrexed and platinum for previously untreated metastatic nonsquamous non-Small-Cell lung cancer. J Clin Oncol (2020) 38(14):1505–17. doi: 10.1200/JCO.19.03136 32150489

[42] Gettinger SN Redman MW Bazhenova L Hirsch FR Mack PC Schwartz LH . Nivolumab plus ipilimumab vs nivolumab for previously treated patients with stage IV squamous cell lung cancer: The lung-MAP S1400I phase 3 randomized clinical trial. JAMA Oncol (2021) 7(9):1368–77. doi: 10.1001/jamaoncol.2021.2209 PMC8283667 34264316

[43] Goldman JW Dvorkin M Chen Y Reinmuth N Hotta K Trukhin D . Durvalumab, with or without tremelimumab, plus platinum-etoposide versus platinum-etoposide alone in first-line treatment of extensive-stage small-cell lung cancer (CASPIAN): updated results from a randomised, controlled, open-label, phase 3 trial. Lancet Oncol (2021) 22(1):51–65. doi: 10.1016/S1470-2045(20)30539-8 33285097

[44] Herbst RS Giaccone G de Marinis F Reinmuth N Vergnenegre A Barrios CH . Atezolizumab for first-line treatment of PD-L1-Selected patients with NSCLC. N Engl J Med (2020) 383(14):1328–39. doi: 10.1056/NEJMoa1917346 32997907

[45] Herbst RS Garon EB Kim DW Cho BC Perez-Gracia JL Han JY . Long-term outcomes and retreatment among patients with previously treated, programmed death-ligand 1−Positive, advanced Non−Small-cell lung cancer in the KEYNOTE-010 study. J Clin Oncol (2020) 38(14):1580–90. doi: 10.1200/JCO.19.02446 32078391

[46] Horn L Mansfield AS Szczęsna A Havel L Krzakowski M Hochmair MJ . First-line atezolizumab plus chemotherapy in extensive-stage small-cell lung cancer. N Engl J Med (2018) 379(23):2220–9. doi: 10.1056/NEJMoa1809064 30280641

[47] Mok TSK Wu YL Kudaba I Kowalski DM Cho BC Turna HZ . Pembrolizumab versus chemotherapy for previously untreated, PD-L1-expressing, locally advanced or metastatic non-small-cell lung cancer (KEYNOTE-042): a randomised, open-label, controlled, phase 3 trial. Lancet (2019) 393(10183):1819–30. doi: 10.1016/S0140-6736(18)32409-7 30955977

[48] Nishio M Barlesi F West H Ball S Bordoni R Cobo M . Atezolizumab plus chemotherapy for first-line treatment of nonsquamous NSCLC: Results from the randomized phase 3 IMpower132 trial. J Thorac Oncol (2021) 16(4):653–64. doi: 10.1016/j.jtho.2020.11.025 33333328

[49] Owonikoko TK Park K Govindan R Ready N Reck M Peters S . Nivolumab and ipilimumab as maintenance therapy in extensive-disease small-cell lung cancer: CheckMate 451. J Clin Oncol (2021) 39(12):1349–59. doi: 10.1200/JCO.20.02212 PMC8078251 33683919

[50] Paz-Ares L Vicente D Tafreshi A Robinson A Soto Parra H Mazières J . A randomized, placebo-controlled trial of pembrolizumab plus chemotherapy in patients with metastatic squamous NSCLC: Protocol-specified final analysis of KEYNOTE-407. J Thorac Oncol (2020) 15(10):1657–69. doi: 10.1016/j.jtho.2020.06.015 32599071

[51] Reck M Rodríguez-Abreu D Robinson AG Hui R Csőszi T Fülöp A . Updated analysis of KEYNOTE-024: Pembrolizumab versus platinum-based chemotherapy for advanced non-Small-Cell lung cancer with PD-L1 tumor proportion score of 50% or greater. J Clin Oncol (2019) 37(7):537–46. doi: 10.1200/JCO.18.00149 30620668

[52] Spigel DR Vicente D Ciuleanu TE Gettinger S Peters S Horn L . Second-line nivolumab in relapsed small-cell lung cancer: CheckMate 331(☆). Ann Oncol (2021) 32(5):631–41. doi: 10.1016/j.annonc.2021.01.071 33539946

[53] West H McCleod M Hussein M Morabito A Rittmeyer A Conter HJ . Atezolizumab in combination with carboplatin plus nab-paclitaxel chemotherapy compared with chemotherapy alone as first-line treatment for metastatic non-squamous non-small-cell lung cancer (IMpower130): a multicentre, randomised, open-label, phase 3 trial. Lancet Oncol (2019) 20(7):924–37. doi: 10.1016/S1470-2045(19)30167-6 31122901

[54] Wu YL Lu S Cheng Y Zhou C Wang J Mok T . Nivolumab versus docetaxel in a predominantly Chinese patient population with previously treated advanced NSCLC: CheckMate 078 randomized phase III clinical trial. J Thorac Oncol (2019) 14(5):867–75. doi: 10.1016/j.jtho.2019.01.006 30659987

[55] Yang Y Wang Z Fang J Yu Q Han B Cang S . Efficacy and safety of sintilimab plus pemetrexed and platinum as first-line treatment for locally advanced or metastatic nonsquamous NSCLC: a randomized, double-blind, phase 3 study (Oncology pRogram by InnovENT anti-PD-1-11). J Thorac Oncol (2020) 15(10):1636–46. doi: 10.1016/j.jtho.2020.07.014 32781263

[56] Hodi FS O'Day SJ McDermott DF Weber RW Sosman JA Haanen JB . Improved survival with ipilimumab in patients with metastatic melanoma. N Engl J Med (2010) 363(8):711–23. doi: 10.1056/NEJMoa1003466 PMC3549297 20525992

[57] Sharma P Allison JP . The future of immune checkpoint therapy. Science (2015) 348(6230):56–61. doi: 10.1126/science.aaa8172 25838373

[58] Sun JY Lu XJ . Cancer immunotherapy: Current applications and challenges. Cancer Lett (2020) 480:1–3. doi: 10.1016/j.canlet.2020.03.024 32229188

[59] Pardoll DM . The blockade of immune checkpoints in cancer immunotherapy. Nat Rev Cancer (2012) 12(4):252–64. doi: 10.1038/nrc3239 PMC4856023 22437870

[60] Socinski MA Jotte RM Cappuzzo F Orlandi F Stroyakovskiy D Nogami N . Atezolizumab for first-line treatment of metastatic nonsquamous NSCLC. N Engl J Med (2018) 378(24):2288–301. doi: 10.1056/NEJMoa1716948 29863955

[61] Paz-Ares L Ciuleanu TE Cobo M Schenker M Zurawski B Menezes J . First-line nivolumab plus ipilimumab combined with two cycles of chemotherapy in patients with non-small-cell lung cancer (CheckMate 9LA): an international, randomised, open-label, phase 3 trial. Lancet Oncol (2021) 22(2):198–211. doi: 10.1016/S1470-2045(20)30641-0 33476593

[62] Paz-Ares L Dvorkin M Chen Y Reinmuth N Hotta K Trukhin D . Durvalumab plus platinum-etoposide versus platinum-etoposide in first-line treatment of extensive-stage small-cell lung cancer (CASPIAN): a randomised, controlled, open-label, phase 3 trial. Lancet (2019) 394(10212):1929–39. doi: 10.1016/S0140-6736(19)32222-6 31590988

[63] Kroschinsky F Stölzel F von Bonin S Beutel G Kochanek M Kiehl M . New drugs, new toxicities: Severe side effects of modern targeted and immunotherapy of cancer and their management. Crit Care (2017) 21(1):89. doi: 10.1186/s13054-017-1678-1 28407743 PMC5391608

[64] Haanen J Carbonnel F Robert C Kerr KM Peters S Larkin J . Management of toxicities from immunotherapy: ESMO clinical practice guidelines for diagnosis, treatment and follow-up. Ann Oncol (2017) 28(suppl_4):iv119–iv42. doi: 10.1093/annonc/mdx225 28881921

[65] Chhabra N Kennedy J . A review of cancer immunotherapy toxicity: Immune checkpoint inhibitors. J Med Toxicol (2021) 17(4):411–24. doi: 10.1007/s13181-021-00833-8 PMC8455777 33826117

[66] Macdonald JB Macdonald B Golitz LE LoRusso P Sekulic A . Cutaneous adverse effects of targeted therapies: Part II: Inhibitors of intracellular molecular signaling pathways. J Am Acad Dermatol (2015) 72(2):221–36. doi: 10.1016/j.jaad.2014.07.033 25592339

[67] Yang S Asnani A . Cardiotoxicities associated with immune checkpoint inhibitors. Curr Probl Cancer (2018) 42(4):422–32. doi: 10.1016/j.currproblcancer.2018.07.002 30173878

[68] Ganatra S Parikh R Neilan TG . Cardiotoxicity of immune therapy. Cardiol Clin (2019) 37(4):385–97. doi: 10.1016/j.ccl.2019.07.008 31587780

[69] Lal JC Brown SA Collier P Cheng F . A retrospective analysis of cardiovascular adverse events associated with immune checkpoint inhibitors. Cardiooncology (2021) 7(1):19. doi: 10.1186/s40959-021-00106-x 34049595 PMC8161966

[70] Puzanov I Diab A Abdallah K Bingham CO Brogdon C Dadu R . Managing toxicities associated with immune checkpoint inhibitors: consensus recommendations from the society for immunotherapy of cancer (SITC) toxicity management working group. J Immunother Cancer (2017) 5(1):95. doi: 10.1186/s40425-017-0300-z 29162153 PMC5697162

[71] Hryniewicki AT Wang C Shatsky RA Coyne CJ . Management of immune checkpoint inhibitor toxicities: A review and clinical guideline for emergency physicians. J Emerg Med (2018) 55(4):489–502. doi: 10.1016/j.jemermed.2018.07.005 30120013

[72] Fessas P Possamai LA Clark J Daniels E Gudd C Mullish BH . Immunotoxicity from checkpoint inhibitor therapy: clinical features and underlying mechanisms. Immunology (2020) 159(2):167–77. doi: 10.1111/imm.13141 PMC6954715 31646612

[73] Yuan H Duan DD Zhang YJ . Comprehensive analysis of treatment-related adverse events of immunotherapy in advanced gastric or gastroesophageal junction cancer: A meta-analysis of randomized controlled trials. Clin Res Hepatol Gastroenterol (2022) 46(10):102031. doi: 10.1016/j.clinre.2022.102031 36261109

[74] Wu Z Chen Q Qu L Li M Wang L Mir MC . Adverse events of immune checkpoint inhibitors therapy for urologic cancer patients in clinical trials: A collaborative systematic review and meta-analysis. Eur Urol (2022) 81(4):414–25. doi: 10.1016/j.eururo.2022.01.028 35101302

[75] Okada N Matsuoka R Sakurada T Goda M Chuma M Yagi K . Risk factors of immune checkpoint inhibitor-related interstitial lung disease in patients with lung cancer: a single-institution retrospective study. Sci Rep (2020) 10(1):13773. doi: 10.1038/s41598-020-70743-2 32792640 PMC7426925

[76] Suzuki Y Karayama M Uto T Fujii M Matsui T Asada K . Assessment of immune-related interstitial lung disease in patients with NSCLC treated with immune checkpoint inhibitors: A multicenter prospective study. J Thorac Oncol (2020) 15(8):1317–27. doi: 10.1016/j.jtho.2020.04.002 32289515

[77] Liu V White DA Zakowski MF Travis W Kris MG Ginsberg MS . Pulmonary toxicity associated with erlotinib. Chest (2007) 132(3):1042–4. doi: 10.1378/chest.07-0050 17873198

[78] Konishi J Yamazaki K Kinoshita I Isobe H Ogura S Sekine S . Analysis of the response and toxicity to gefitinib of non-small cell lung cancer. Anticancer Res (2005) 25(1B):435–41. 15816608

[79] Grande C Villanueva MJ Huidobro G Casal J . Docetaxel-induced interstitial pneumonitis following non-small-cell lung cancer treatment. Clin Transl Oncol (2007) 9(9):578–81. doi: 10.1007/s12094-007-0106-4 17921105

[80] Roychowdhury DF Cassidy CA Peterson P Arning M . A report on serious pulmonary toxicity associated with gemcitabine-based therapy. Invest New Drugs (2002) 20(3):311–5. doi: 10.1023/A:1016214032272 12201493

[81] Hochstrasser A Benz G Joerger M Templeton A Brutsche M Früh M . Interstitial pneumonitis after treatment with pemetrexed: A rare event? Chemotherapy (2012) 58(1):84–8. doi: 10.1159/000336131 22377772

[82] Loriot Y Ferte C Gomez-Roca C Moldovan C Bahleda R Wislez M . Pemetrexed-induced pneumonitis: A case report. Clin Lung Cancer (2009) 10(5):364–6. doi: 10.3816/CLC.2009.n.050 19808196

[83] Créquit P Wislez M Feith JF Rozensztajn N Jabot L Friard S . Crizotinib associated with ground-glass opacity predominant pattern interstitial lung disease: A retrospective observational cohort study with a systematic literature review. J Thorac Oncol (2015) 10(8):1148–55. doi: 10.1097/JTO.0000000000000577 26200268

[84] Yoshino K Nakayama T Ito A Sato E Kitano S . Severe colitis after PD-1 blockade with nivolumab in advanced melanoma patients: Potential role of Th1-dominant immune response in immune-related adverse events: two case reports. BMC Cancer (2019) 19(1):1019. doi: 10.1186/s12885-019-6138-7 31664934 PMC6819390

[85] Lim SY Lee JH Gide TN Menzies AM Guminski A Carlino MS . Circulating cytokines predict immune-related toxicity in melanoma patients receiving anti-PD-1-Based immunotherapy. Clin Cancer Res (2019) 25(5):1557–63. doi: 10.1158/1078-0432.CCR-18-2795 30409824

[86] Tanaka R Okiyama N Okune M Ishitsuka Y Watanabe R Furuta J . Serum level of interleukin-6 is increased in nivolumab-associated psoriasiform dermatitis and tumor necrosis factor-α is a biomarker of nivolumab recativity. J Dermatol Sci (2017) 86(1):71–3. doi: 10.1016/j.jdermsci.2016.12.019 28069323

[87] Abraham C Cho J . Interleukin-23/Th17 pathways and inflammatory bowel disease. Inflammation Bowel Dis (2009) 15(7):1090–100. doi: 10.1002/ibd.20894 19253307

[88] Tarhini AA Zahoor H Lin Y Malhotra U Sander C Butterfield LH . Baseline circulating IL-17 predicts toxicity while TGF-β1 and IL-10 are prognostic of relapse in ipilimumab neoadjuvant therapy of melanoma. J Immunother Cancer (2015) 3:39. doi: 10.1186/s40425-015-0081-1 26380086 PMC4570556

[89] Friedman CF Proverbs-Singh TA Postow MA . Treatment of the immune-related adverse effects of immune checkpoint inhibitors: A review. JAMA Oncol (2016) 2(10):1346–53. doi: 10.1001/jamaoncol.2016.1051 27367787

[90] Grim-Stieger M Keilani M Mader RM Marosi C Schmidinger M Zielinski CC . Serum levels of tumour necrosis factor-alpha and interleukin-6 and their correlation with body mass index, weight loss, appetite and survival rate–preliminary data of Viennese outpatients with metastatic cancer during palliative chemotherapy. Eur J Cancer Care (Engl) (2008) 17(5):454–62. doi: 10.1111/j.1365-2354.2007.00874.x 18637115

[91] Penna F Minero VG Costamagna D Bonelli G Baccino FM Costelli P . Anti-cytokine strategies for the treatment of cancer-related anorexia and cachexia. Expert Opin Biol Ther (2010) 10(8):1241–50. doi: 10.1517/14712598.2010.503773 20594117

[92] Delaunay M Cadranel J Lusque A Meyer N Gounant V Moro-Sibilot D . Immune-checkpoint inhibitors associated interstitial Lung Dis Cancer patients. Eur Respir J (2017) 50(2):1700050. doi: 10.1183/13993003.00050-2017 28798088

[93] Shen CI Yeh YC Chiu CH . Progressive pleural effusion as an immune-related adverse event in NSCLC: A case report. JTO Clin Res Rep (2021) 2(5):100156. doi: 10.1016/j.jtocrr.2021.100156 34590016 PMC8474266

[94] Sawada R Matsui Y Uchino J Okura N Morimoto Y Iwasaku M . Late-onset pleural and pericardial effusion as immune-related adverse events after 94 cycles of nivolumab. Intern Med (2021) 60(22):3585–8. doi: 10.2169/internalmedicine.7219-21 PMC8666223 34092733

[95] Kolla BC Patel MR . Recurrent pleural effusions and cardiac tamponade as possible manifestations of pseudoprogression associated with nivolumab therapy- a report of two cases. J Immunother Cancer (2016) 4:80. doi: 10.1186/s40425-016-0185-2 27895919 PMC5109681

[96] Rittmeyer A Barlesi F Waterkamp D Park K Ciardiello F von Pawel J . Atezolizumab versus docetaxel in patients with previously treated non-small-cell lung cancer (OAK): a phase 3, open-label, multicentre randomised controlled trial. Lancet (2017) 389(10066):255–65. doi: 10.1016/S0140-6736(16)32517-X PMC6886121 27979383

[97] Fehrenbacher L Spira A Ballinger M Kowanetz M Vansteenkiste J Mazieres J . Atezolizumab versus docetaxel for patients with previously treated non-small-cell lung cancer (POPLAR): A multicentre, open-label, phase 2 randomised controlled trial. Lancet (2016) 387(10030):1837–46. doi: 10.1016/S0140-6736(16)00587-0 26970723

[98] Antonia SJ Villegas A Daniel D Vicente D Murakami S Hui R . Durvalumab after chemoradiotherapy in stage III non-Small-Cell lung cancer. N Engl J Med (2017) 377(20):1919–29. doi: 10.1056/NEJMoa1709937 28885881

[99] Ferris RL Blumenschein G Jr. Fayette J Guigay J Colevas AD Licitra L . Nivolumab for recurrent squamous-cell carcinoma of the head and neck. N Engl J Med (2016) 375(19):1856–67. doi: 10.1056/NEJMoa1602252 PMC5564292 27718784

[100] Weber JS D'Angelo SP Minor D Hodi FS Gutzmer R Neyns B . Nivolumab versus chemotherapy in patients with advanced melanoma who progressed after anti-CTLA-4 treatment (CheckMate 037): A randomised, controlled, open-label, phase 3 trial. Lancet Oncol (2015) 16(4):375–84. doi: 10.1016/S1470-2045(15)70076-8 25795410

[101] Robert C Long GV Brady B Dutriaux C Maio M Mortier L . Nivolumab in previously untreated melanoma without BRAF mutation. N Engl J Med (2015) 372(4):320–30. doi: 10.1056/NEJMoa1412082 25399552

[102] Bellmunt J de Wit R Vaughn DJ Fradet Y Lee JL Fong L . Pembrolizumab as second-line therapy for advanced urothelial carcinoma. N Engl J Med (2017) 376(11):1015–26. doi: 10.1056/NEJMoa1613683 PMC5635424 28212060

[103] Ribas A Puzanov I Dummer R Schadendorf D Hamid O Robert C . Pembrolizumab versus investigator-choice chemotherapy for ipilimumab-refractory melanoma (KEYNOTE-002): A randomised, controlled, phase 2 trial. Lancet Oncol (2015) 16(8):908–18. doi: 10.1016/S1470-2045(15)00083-2 PMC9004487 26115796

[104] Maio M Scherpereel A Calabrò L Aerts J Perez SC Bearz A . Tremelimumab as second-line or third-line treatment in relapsed malignant mesothelioma (DETERMINE): A multicentre, international, randomised, double-blind, placebo-controlled phase 2b trial. Lancet Oncol (2017) 18(9):1261–73. doi: 10.1016/S1470-2045(17)30446-1 28729154

[105] Eggermont AM Chiarion-Sileni V Grob JJ Dummer R Wolchok JD Schmidt H . Adjuvant ipilimumab versus placebo after complete resection of high-risk stage III melanoma (EORTC 18071): A randomised, double-blind, phase 3 trial. Lancet Oncol (2015) 16(5):522–30. doi: 10.1016/S1470-2045(15)70122-1 25840693

[106] Kwon ED Drake CG Scher HI Fizazi K Bossi A van den Eertwegh AJ . Ipilimumab versus placebo after radiotherapy in patients with metastatic castration-resistant prostate cancer that had progressed after docetaxel chemotherapy (CA184-043): a multicentre, randomised, double-blind, phase 3 trial. Lancet Oncol (2014) 15(7):700–12. doi: 10.1016/S1470-2045(14)70189-5 PMC4418935 24831977

[107] Govindan R Szczesna A Ahn MJ Schneider CP Gonzalez Mella PF Barlesi F . Phase III trial of ipilimumab combined with paclitaxel and carboplatin in advanced squamous non-Small-Cell lung cancer. J Clin Oncol (2017) 35(30):3449–57. doi: 10.1200/JCO.2016.71.7629 28854067

[108] Robert C Thomas L Bondarenko I O'Day S Weber J Garbe C . Ipilimumab plus dacarbazine for previously untreated metastatic melanoma. N Engl J Med (2011) 364(26):2517–26. doi: 10.1056/NEJMoa1104621 21639810

[109] Langer CJ Gadgeel SM Borghaei H Papadimitrakopoulou VA Patnaik A Powell SF . Carboplatin and pemetrexed with or without pembrolizumab for advanced, non-squamous non-small-cell lung cancer: A randomised, phase 2 cohort of the open-label KEYNOTE-021 study. Lancet Oncol (2016) 17(11):1497–508. doi: 10.1016/S1470-2045(16)30498-3 PMC6886237 27745820

[110] Wolchok JD Chiarion-Sileni V Gonzalez R Rutkowski P Grob JJ Cowey CL . Overall survival with combined nivolumab and ipilimumab in advanced melanoma. N Engl J Med (2017) 377(14):1345–56. doi: 10.1056/NEJMoa1709684 PMC5706778 28889792

[111] Antonia SJ López-Martin JA Bendell J Ott PA Taylor M Eder JP . Nivolumab alone and nivolumab plus ipilimumab in recurrent small-cell lung cancer (CheckMate 032): a multicentre, open-label, phase 1/2 trial. Lancet Oncol (2016) 17(7):883–95. doi: 10.1016/S1470-2045(16)30098-5 27269741

[112] Hodi FS Chesney J Pavlick AC Robert C Grossmann KF McDermott DF . Combined nivolumab and ipilimumab versus ipilimumab alone in patients with advanced melanoma: 2-year overall survival outcomes in a multicentre, randomised, controlled, phase 2 trial. Lancet Oncol (2016) 17(11):1558–68. doi: 10.1016/S1470-2045(16)30366-7 PMC5630525 27622997

[113] Robert C Schachter J Long GV Arance A Grob JJ Mortier L . Pembrolizumab versus ipilimumab in advanced melanoma. N Engl J Med (2015) 372(26):2521–32. doi: 10.1056/NEJMoa1503093 25891173

[114] Chow LQ . Exploring novel immune-related toxicities and endpoints with immune-checkpoint inhibitors in non-small cell lung cancer. Am Soc Clin Oncol Educ Book (2013) 33:e280. doi: 10.14694/EdBook_AM.2013.33.e280 23714523

[115] Nishino M Giobbie-Hurder A Hatabu H Ramaiya NH Hodi FS . Incidence of programmed cell death 1 inhibitor-related pneumonitis in patients with advanced cancer: A systematic review and meta-analysis. JAMA Oncol (2016) 2(12):1607–16. doi: 10.1001/jamaoncol.2016.2453 27540850

[116] Su Q Zhu EC Wu JB Li T Hou YL Wang DY . Risk of pneumonitis and pneumonia associated with immune checkpoint inhibitors for solid tumors: A systematic review and meta-analysis. Front Immunol (2019) 10:108. doi: 10.3389/fimmu.2019.00108 30778352 PMC6369169

[117] Cupp J Culakova E Poniewierski MS Dale DC Lyman GH Crawford J . Analysis of factors associated with in-hospital mortality in lung cancer chemotherapy patients with neutropenia. Clin Lung Cancer (2018) 19(2):e163–e9. doi: 10.1016/j.cllc.2017.10.013 29233611

