# Supplementary figures and images for: Immune checkpoint inhibitors related respiratory disorders in patients with lung cancer: A meta-analysis of randomized controlled trials
Source: Front Immunol. 2023 Feb 28;14:1115305. doi: 10.3389/fimmu.2023.1115305 (PMC10011157; doi:10.3389/fimmu.2023.1115305)

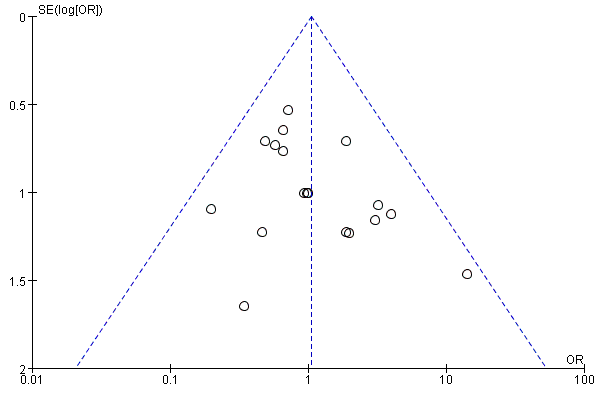

Supplement: Supplementary Figure 1 — The funnel plot for accessing publication bias: chronic obstructive pulmonary diseases. [file Image_1.png]

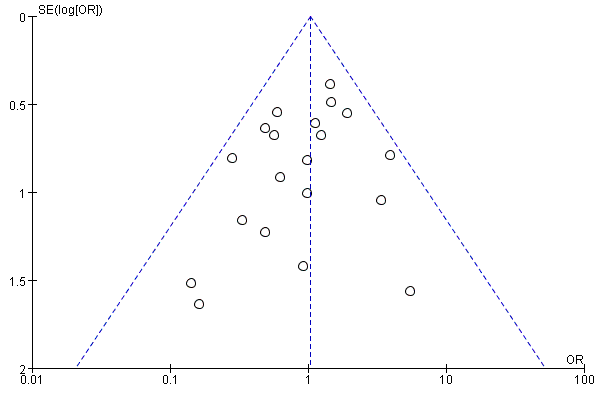

Supplement: Supplementary Figure 2 — The funnel plot for accessing publication bias: dyspnea. [file Image_2.png]

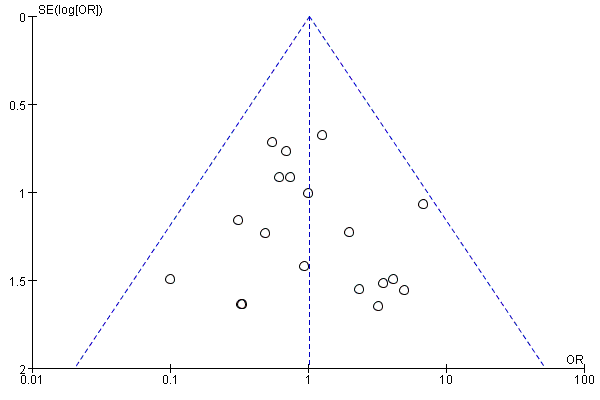

Supplement: Supplementary Figure 3 — The funnel plot for accessing publication bias: hemoptysis. [file Image_3.png]

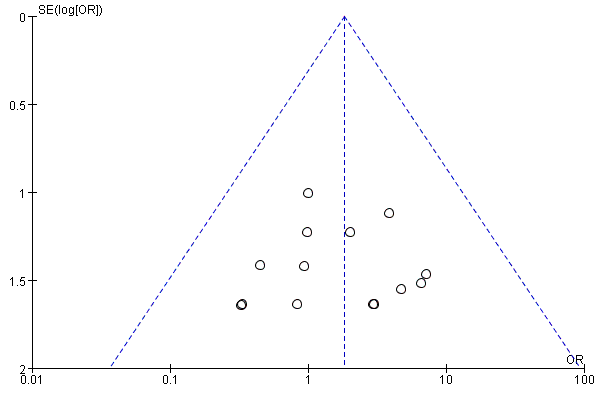

Supplement: Supplementary Figure 4 — The funnel plot for accessing publication bias: interstitial lung disease. [file Image_4.png]

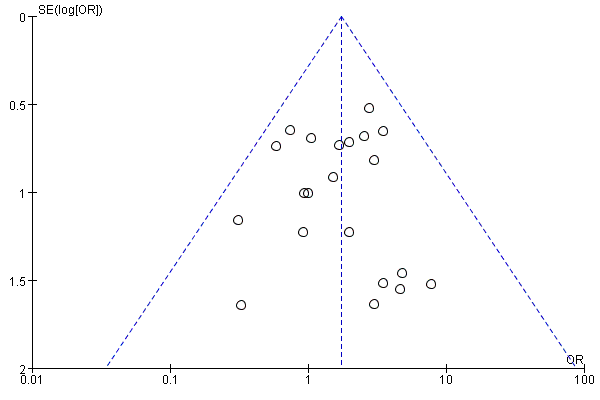

Supplement: Supplementary Figure 5 — The funnel plot for accessing publication bias: pleural effusion. [file Image_5.png]

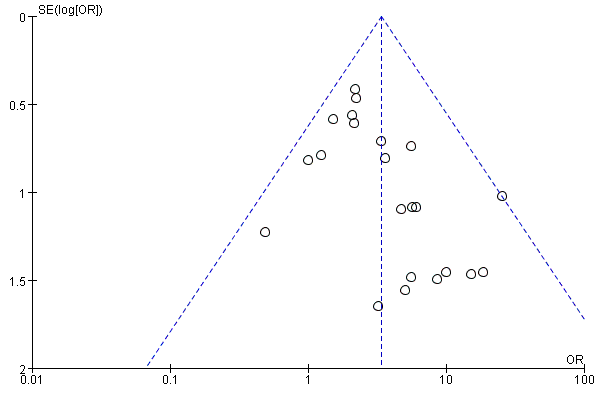

Supplement: Supplementary Figure 6 — The funnel plot for accessing publication bias: pneumonitis. [file Image_6.png]

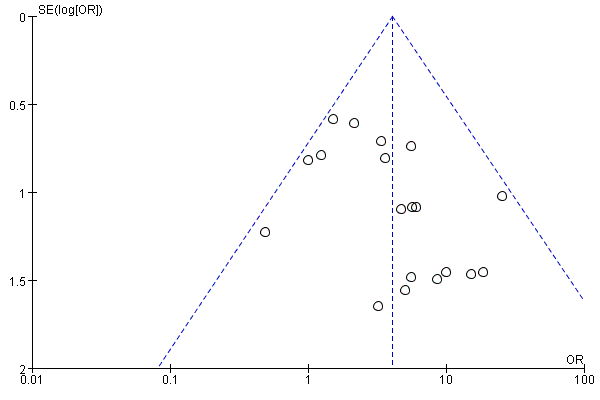

Supplement: Supplementary Figure 7 — The funnel plot for accessing publication bias: pneumothorax. [file Image_7.png]

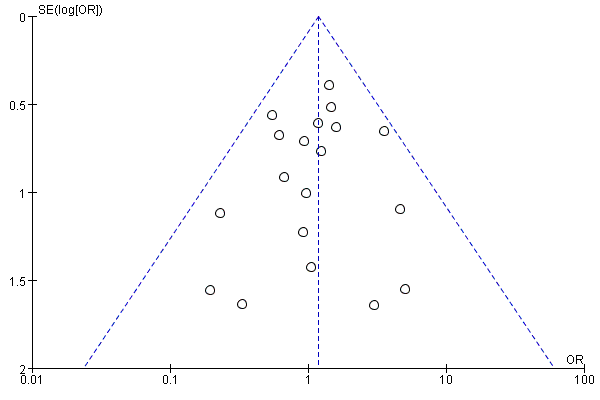

Supplement: Supplementary Figure 8 — The funnel plot for accessing publication bias: pulmonary embolism. [file Image_8.png]

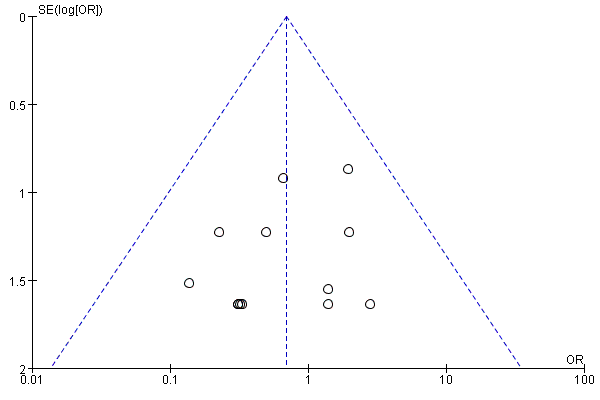

Supplement: Supplementary Figure 9 — The funnel plot for accessing publication bias: pulmonary hemorrhage. [file Image_9.png]

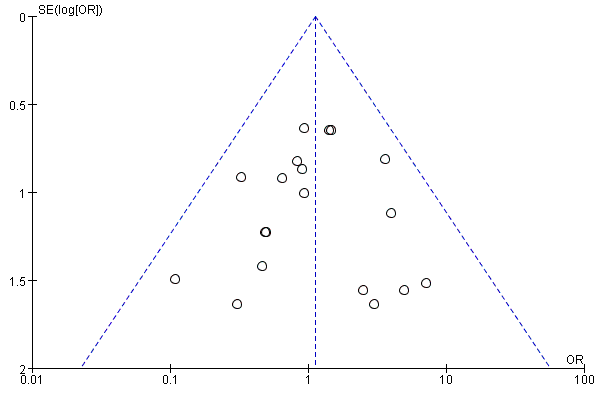

Supplement: Supplementary Figure 10 — The funnel plot for accessing publication bias: respiratory failure. [file Image_10.png]
